# Supplementary material for: Changes in phenology mediate vertebrate population responses to temperature globally
Source: Nat Commun. 2026 Jan 12;17:479. doi: 10.1038/s41467-025-68172-8 (PMC12800269; doi:10.1038/s41467-025-68172-8)
Supplement: Supplementary file 1 — Supplementary Information [file 41467_2025_68172_MOESM1_ESM.pdf]

Supporting Information

Radchuk et al. **Changes in phenology mediate vertebrate population responses to temperature globally.** *Nature Communications*

SUPPLEMENTARY INFORMATION GUIDE

Supplementary Figures (S1-S29) 2

Supplementary Tables (S1-S12) 33

Supplementary Notes 43

References 49

SUPPLEMENTARY FIGURES

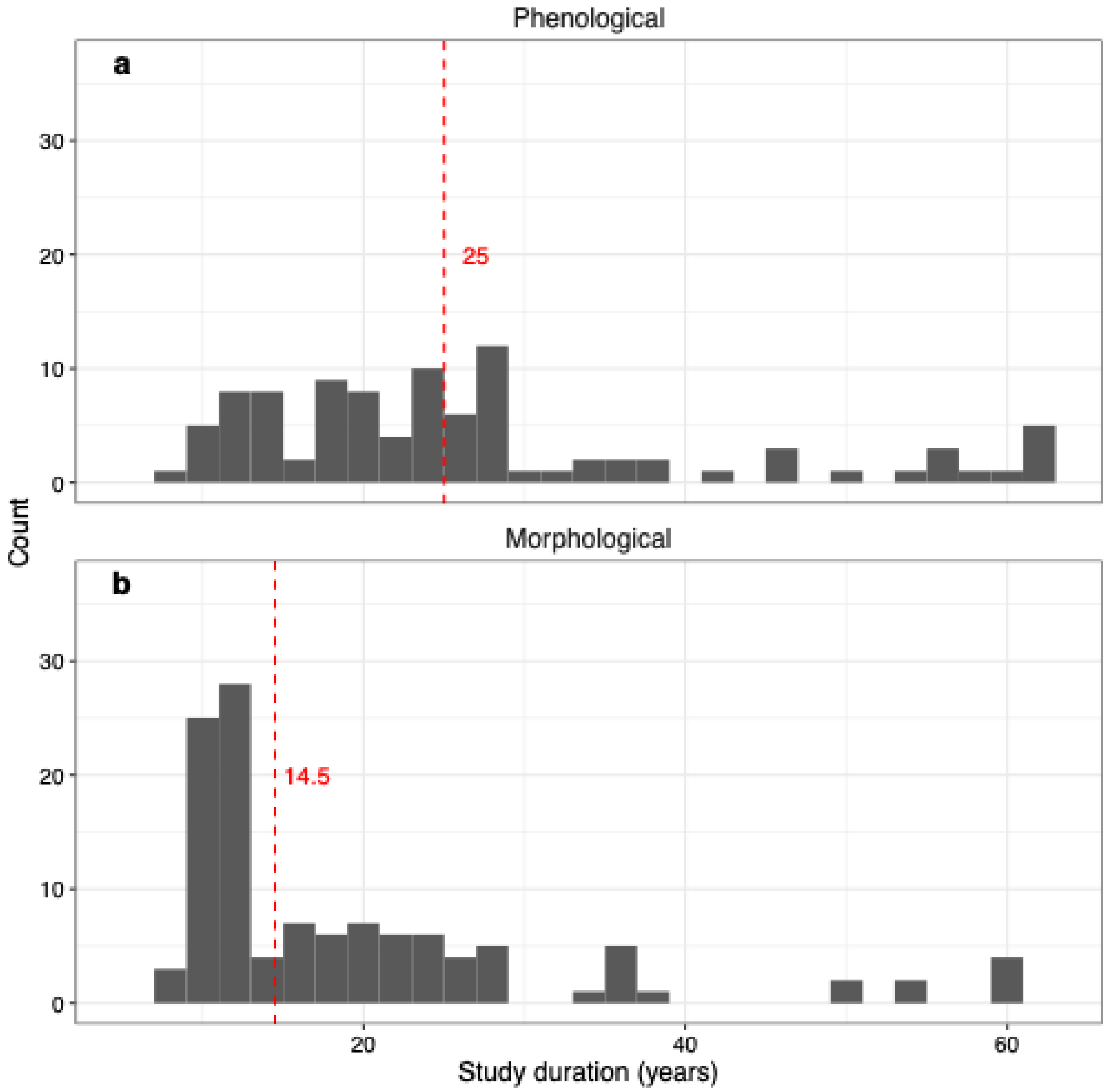

Supplementary Figure S1. Histogram of study durations for the two trait categories. Study durations are shown per trait category: a) phenological and b) morphological traits. The vertical red dashed line and the value in red next to it shows the median study duration.

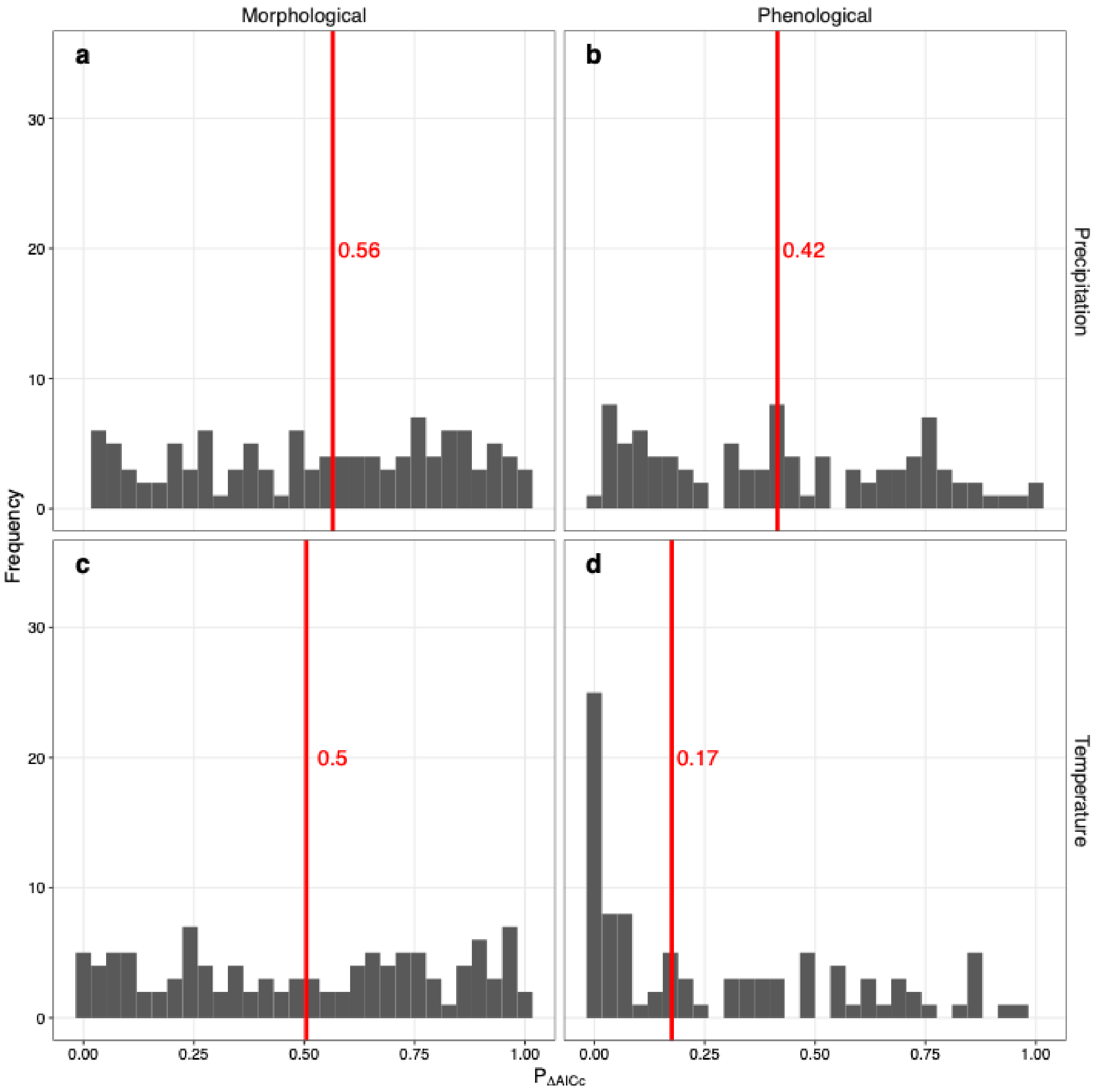

Supplementary Figure S2. Histograms of the probability that the detected climatic signals were spurious ($P_{\Delta AICc}$). This probability was obtained with the randomization procedure in the sliding window analysis for models fitted to morphological (a, c) and phenological traits (b, d) for precipitation (a, b) and temperature (c, d). Vertical red line and the number next to it shows the median $P_{\Delta AICc}$ value for each group.

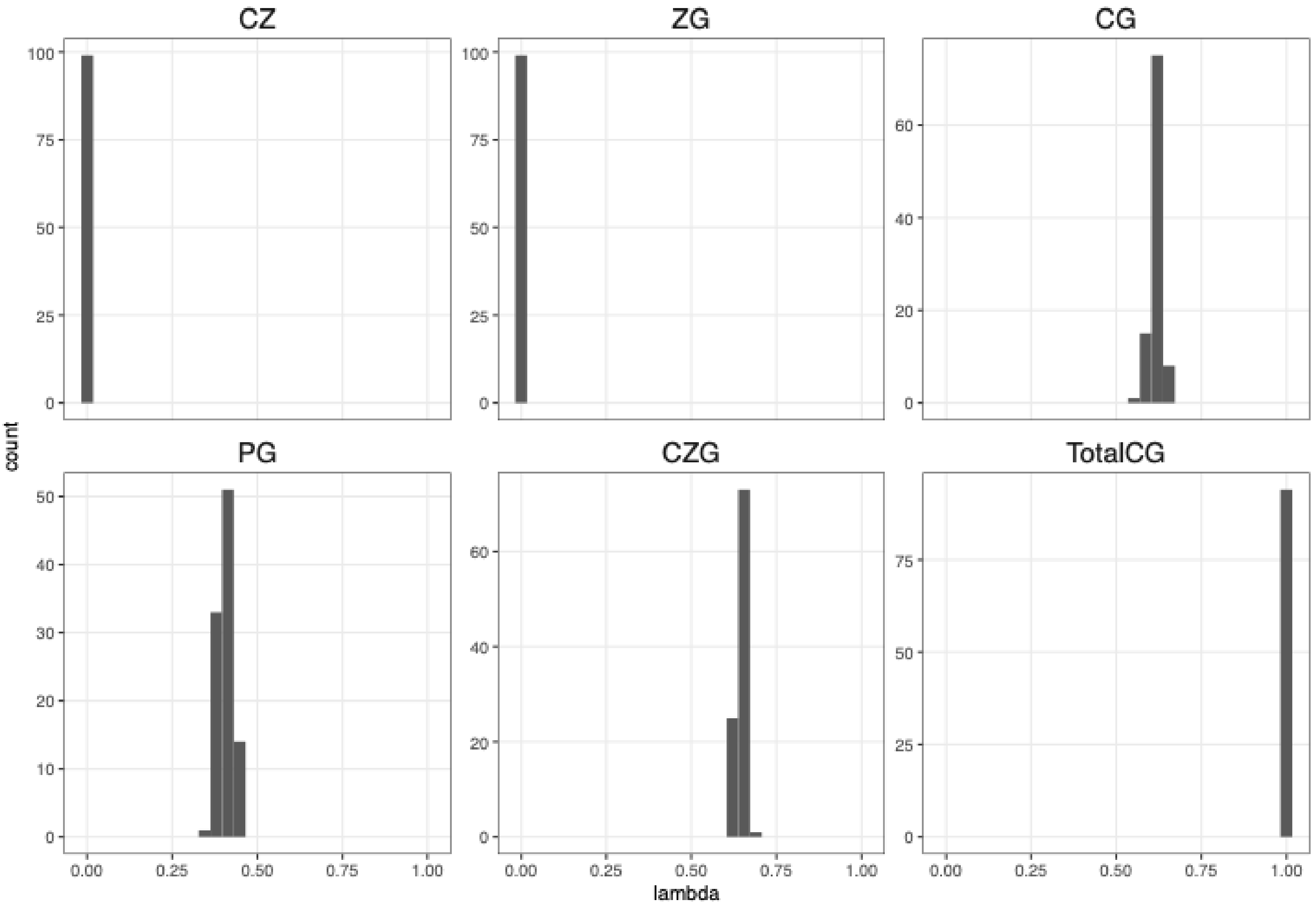

Supplementary Figure S3. Histograms of Pagel's lambdas obtained across 100 models fitted to 100 randomly drawn posterior vertebrate mega-trees. Models are fitted to explain the effect of hypothesized predictors on each path in our conceptual framework. The path is shown above each panel (see Fig. 1 in the main text). These analyses focus on studies that recorded phenological changes in response to temperature.

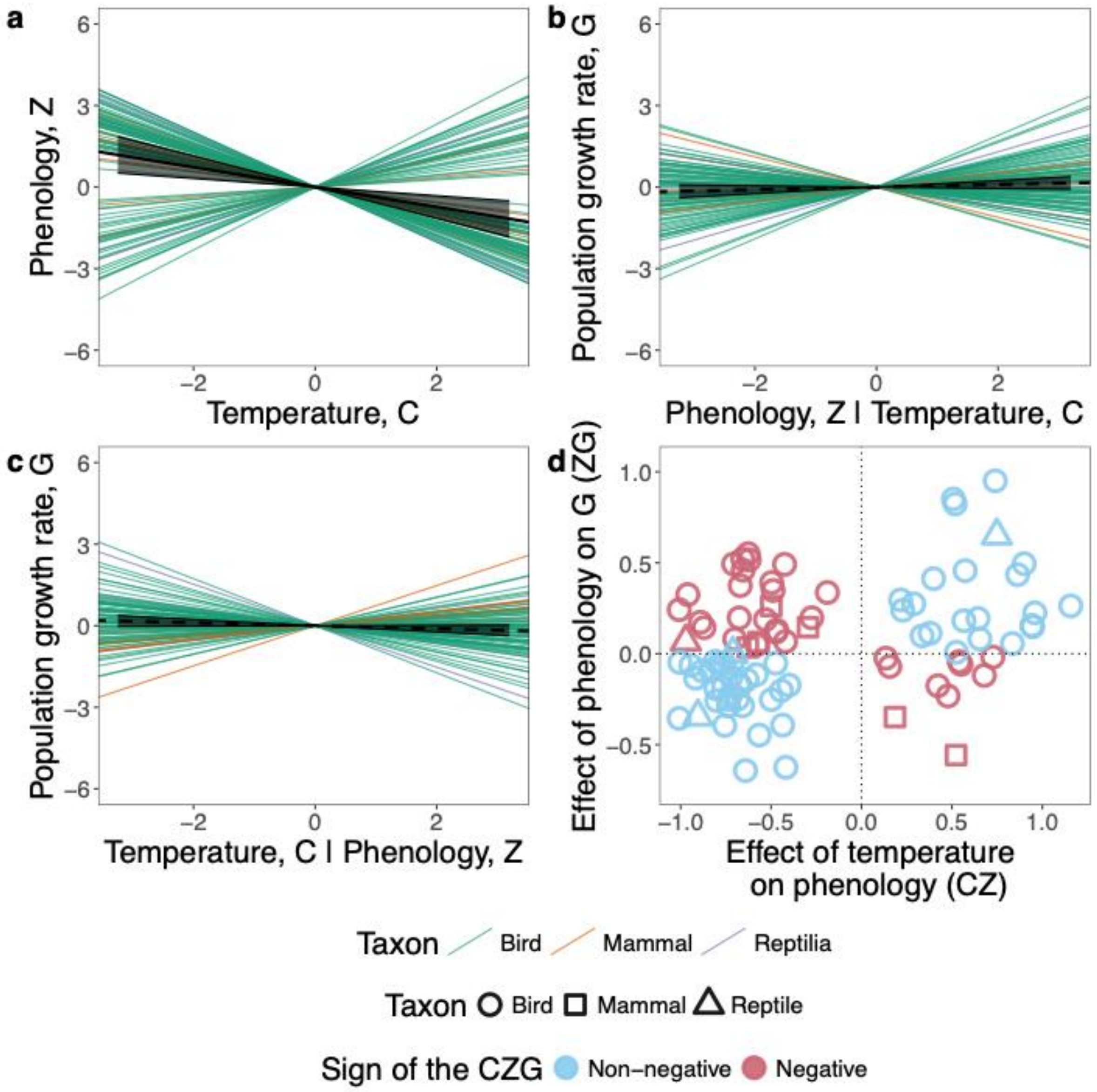

Supplementary Figure S4. Evidence that phenological responses to year-detrended temperature propagated to population growth in most species. Across studies, phenology was earlier in years warmer than average (a), the association between phenology and population growth rate conditional on temperature and population size was not significant (b), the direct effect of temperature on population growth rate (mediated by all other traits but phenology) did not differ from 0 (c) and the proportion of studies with non-negative phenology-mediated effect of temperature on population growth rate (CZG) was significantly higher than expected by chance (d). Thin lines in a), b) and c) show estimated slopes for each single study and are coloured by taxon. Black thick lines show the overall across-study effects. Solid thick lines demonstrate significant effects and dashed lines non-significant ones. The grey shaded bands around the black thick lines are the 95% confidence intervals. In d) studies with non-negative CZG are shown in blue and those with negative CZG – in mauve. The shape of the sign in d) reflects the taxon.

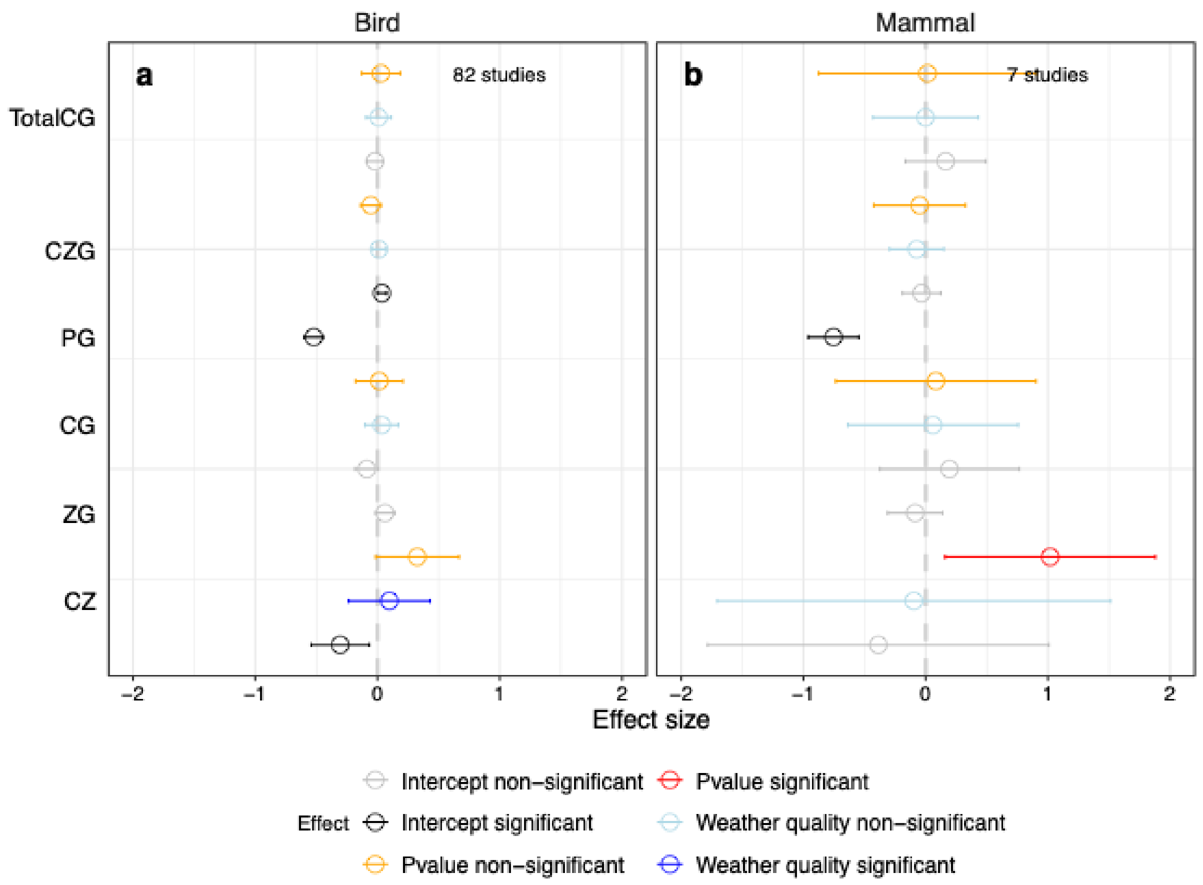

Supplementary Figure S5. Across-study effects obtained for each path in our conceptual framework by applying meta-analyses to phenological changes in response to temperature separately for each taxon. The across-study effects are shown for a) birds and b) mammals. Analyses focus on studies that recorded phenological changes in response to temperature. Bars correspond to the confidence intervals. In addition to the effect sizes reflecting intercepts in our models (black: significant, grey: non-significant), the results on covariates are also shown: $P_{\Delta AICc}$ value from the sliding climate window analyses (red if significant, light orange if non-significant) and weather quality (dark blue if significant, light blue if non-significant). For intercept and p-value the points show their estimates but for the weather quality (which is a qualitative predictor) the difference between the reference level, i.e. “exact” and the “approximate location” level is shown, meaning that the overall effect of the weather quality may be significant even if the depicted confidence intervals overlap with 0. The vertical grey dashed line indicates 0. The significant (determined as CI that do not overlap with 0; this is reflected differently for the weather quality, for which the difference between two levels is visualised) effects are shown by darker shades, whereas the non-significant ones by lighter shades. The numbers of studies for each meta-analysis are given in the upper right corner. The sample sizes are lower than the original ones (birds: 84, mammals: 9; see Fig. 2) because SEMs for 2 studies for each taxon did not converge.

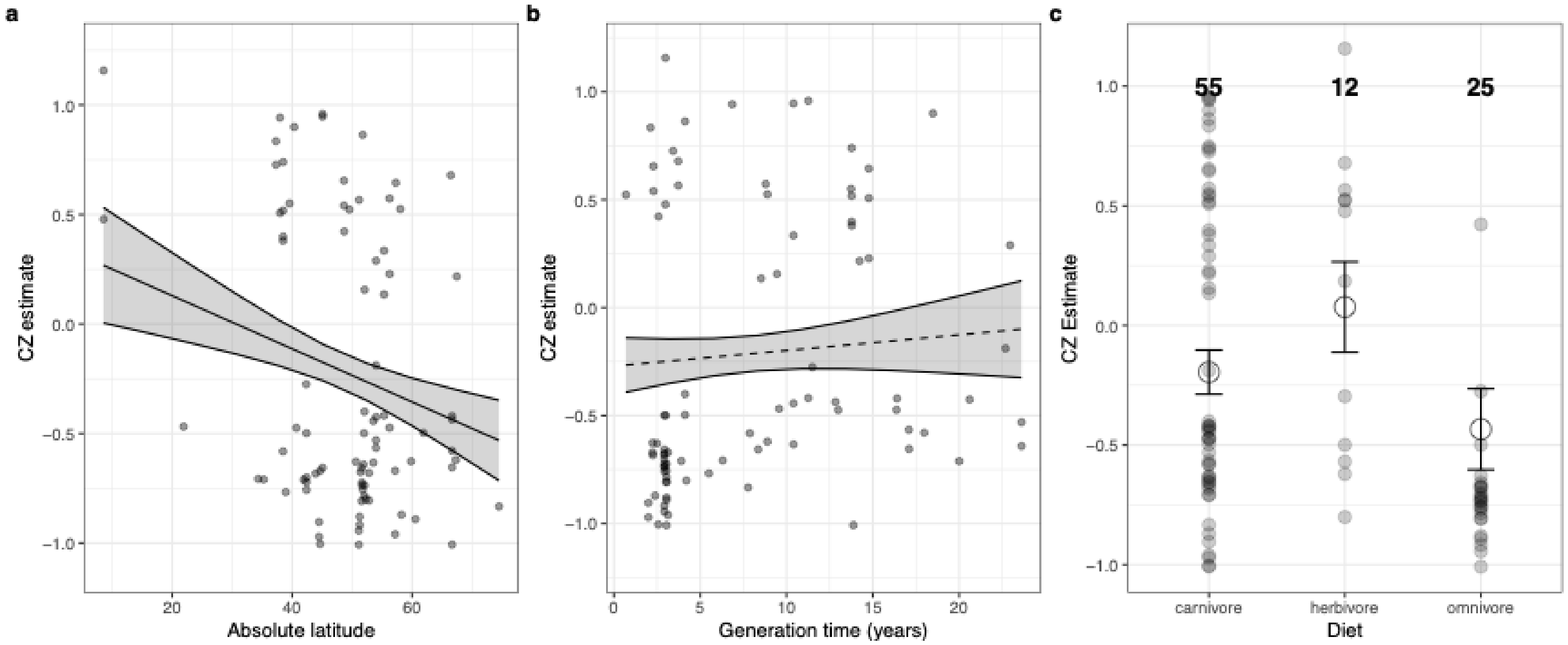

Supplementary Figure S6. Effect of hypothesized covariates on phenological responses of species to temperature$_d$ (denoting henceforth the year-detrended temperature). (a) Phenological responses to temperature are strong (i.e. stronger advancements of phenology) at higher latitudes and they become weaker towards the equator. (b) Generation times are not significantly associated with phenological responses to temperature. (c) The diet is not significantly associated with phenological responses to temperature. Data points show raw data, lines and shaded regions in a) and b) show model fits and +/- 1 standard deviation, respectively; the lines in a) and b) are solid if the association is significant at the $\alpha = 0.05$ level and dashed otherwise. Large points and bars in c) show the model fits and +/- 1 standard deviation, respectively. Numbers above points in c) show sample sizes.

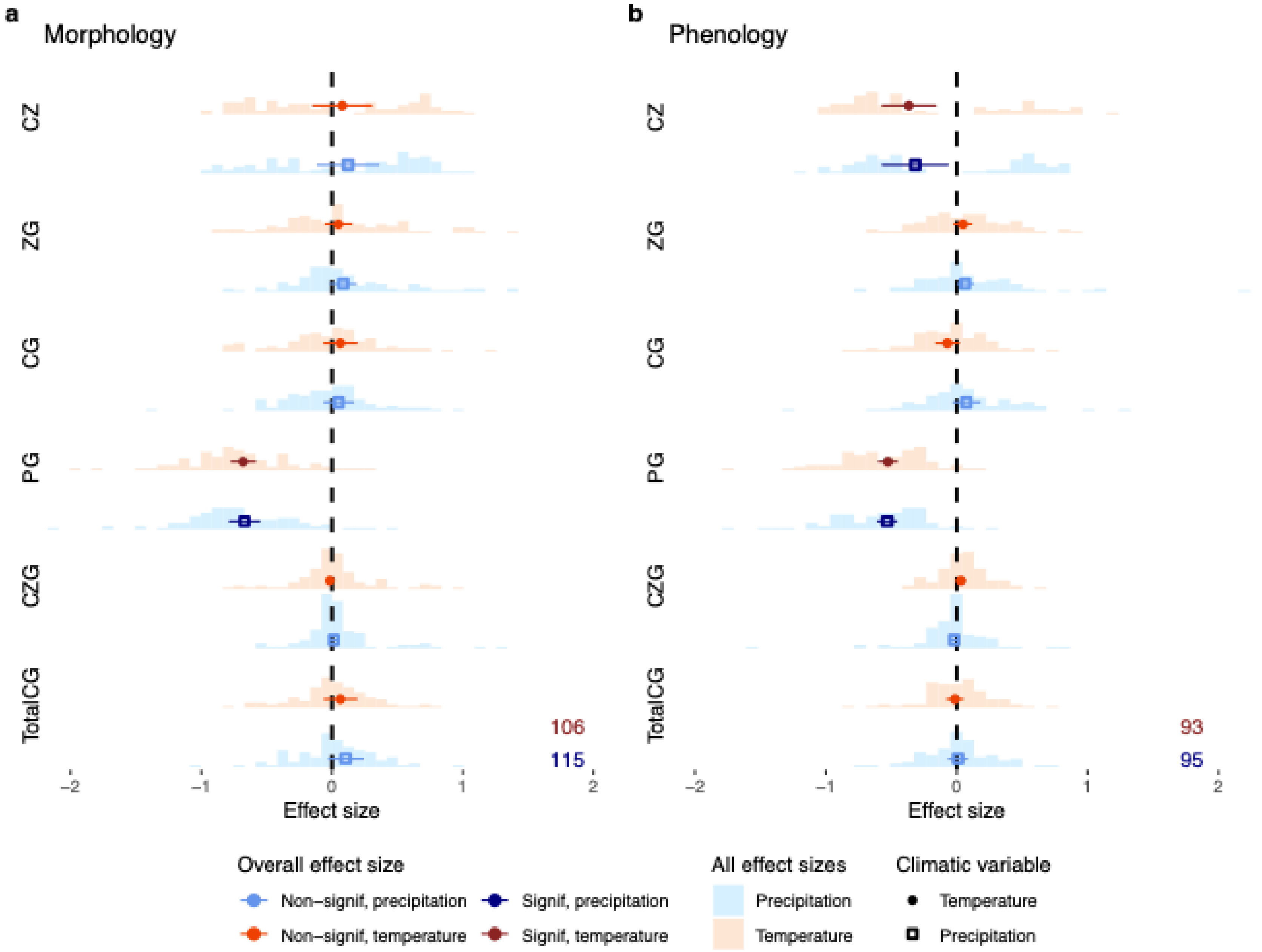
a
Morphology
b
Phenology
CZ
ZG
CG
PG
CZG
TotalCG
106
115
93
95
−2
−1
0
1
2
Effect size
Overall effect size
Non−signif, precipitation
Signif, precipitation
Non−signif, temperature
Signif, temperature
All effect sizes
Precipitation
Temperature
Climatic variable
Temperature
Precipitation

Supplementary Figure S7. Estimated study-specific path coefficients for each relationship in our conceptual framework (Fig. 1a, main text) for each climate variable. Analyses were performed for a) morphological and b) phenological traits separately. Points and bars show the across-study path coefficients and their 95% confidence intervals obtained from the meta-analyses. The notation of relationships is the same as in Fig. 1 in the main text. Significant /non-significant across-study effects are shown in dark red/orange and dark blue/light blue for temperature and precipitation, respectively. The vertical dashed line reflects an effect size of 0. Numbers in the right bottom corner of each panel show the number of studies for temperature (red) and precipitation (blue).

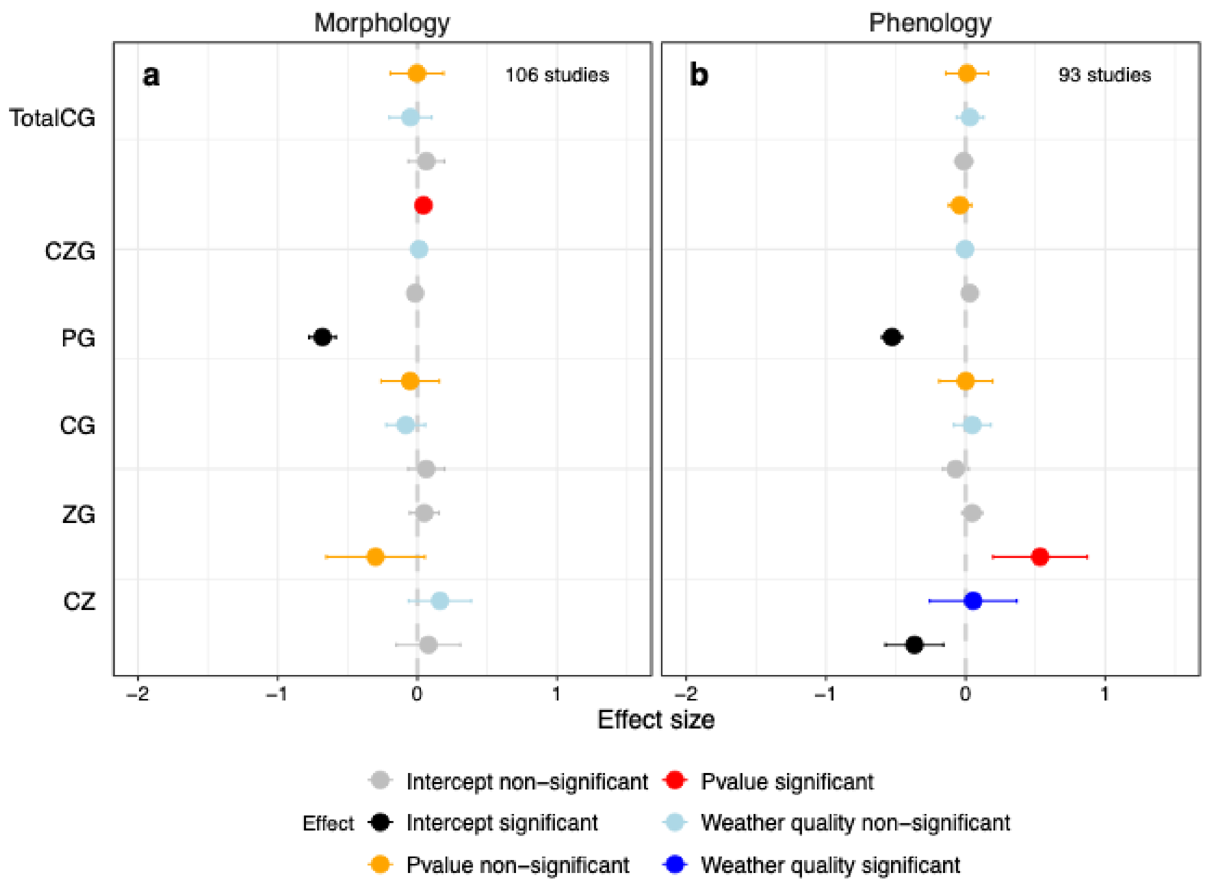

Supplementary Figure S8. For studies focusing on temperature, shown are across-study effects obtained with meta-analyses for each path in the SEM (see Fig. 1 in the main text). Bars correspond to the confidence intervals. Results are shown per trait category: a) morphology and b) phenology. In addition to the effect sizes reflecting intercepts (black: significant, grey: non-significant), the results on covariates are also shown: $P_{\Delta AICc}$ value from the sliding climate window analyses (red if significant, light orange if non-significant) and weather quality (dark blue if significant, light blue if non-significant). For intercept and p-value the points show their estimates but for the weather quality (which is a qualitative predictor) the difference between the reference level, i.e. "exact" and the "approximate location" level is shown, meaning that the overall effect of the weather quality may be significant even if the depicted confidence intervals overlap with 0. The vertical grey dashed line indicates 0. The significant (determined as CI that do not overlap with 0; this is reflected differently for the weather quality, for which the difference between two levels is visualised) effects are shown by darker shades, whereas the non-significant ones by lighter shades. The numbers of studies for each meta-analysis are given in the upper right corner.

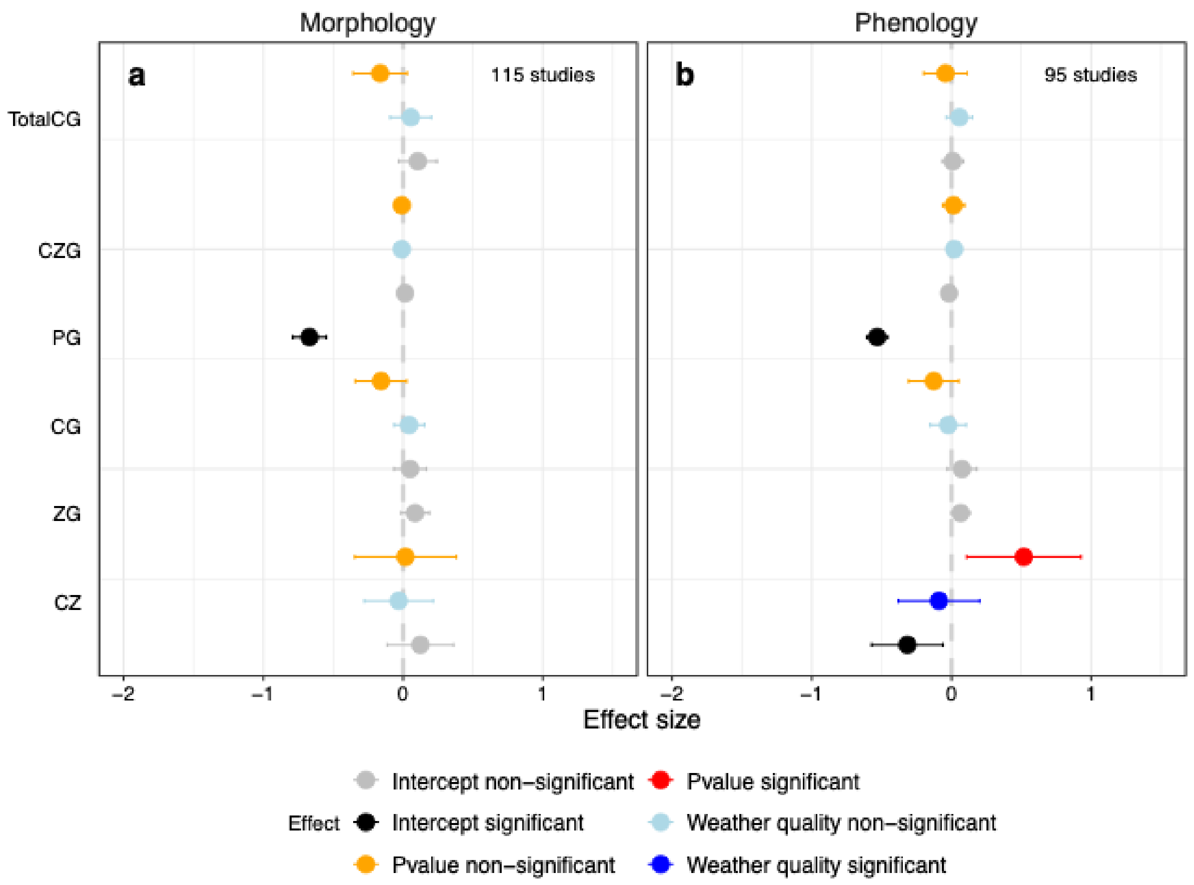

Supplementary Figure S9. For studies focusing on precipitation, shown are across-study effects obtained with meta-analyses for each path in the SEM (see Fig. 1 in the main text). Bars correspond to the confidence intervals. Results are shown per trait category: a) morphology and b) phenology. In addition to the effect sizes reflecting intercepts (black: significant, grey: non-significant), the results on covariates are also shown: $P_{\Delta AICc}$ value from the sliding climate window analyses (red if significant, light orange if non-significant) and weather quality (dark blue if significant, light blue if non-significant). For intercept and p-value, the points show their estimates but for the weather quality (which is a qualitative predictor) the difference between the reference level, i.e. “exact” and the “approximate location” level is shown, meaning that the overall effect of the weather quality may be significant even if the depicted confidence intervals overlap with 0. The vertical grey dashed line indicates 0. The significant (determined as CI that do not overlap with 0; this is reflected differently for the weather quality, for which the difference between two levels is visualised) effects are shown by darker shades, whereas the non-significant ones by lighter shades. Numbers of studies for each meta-analysis are given in the upper right corner.

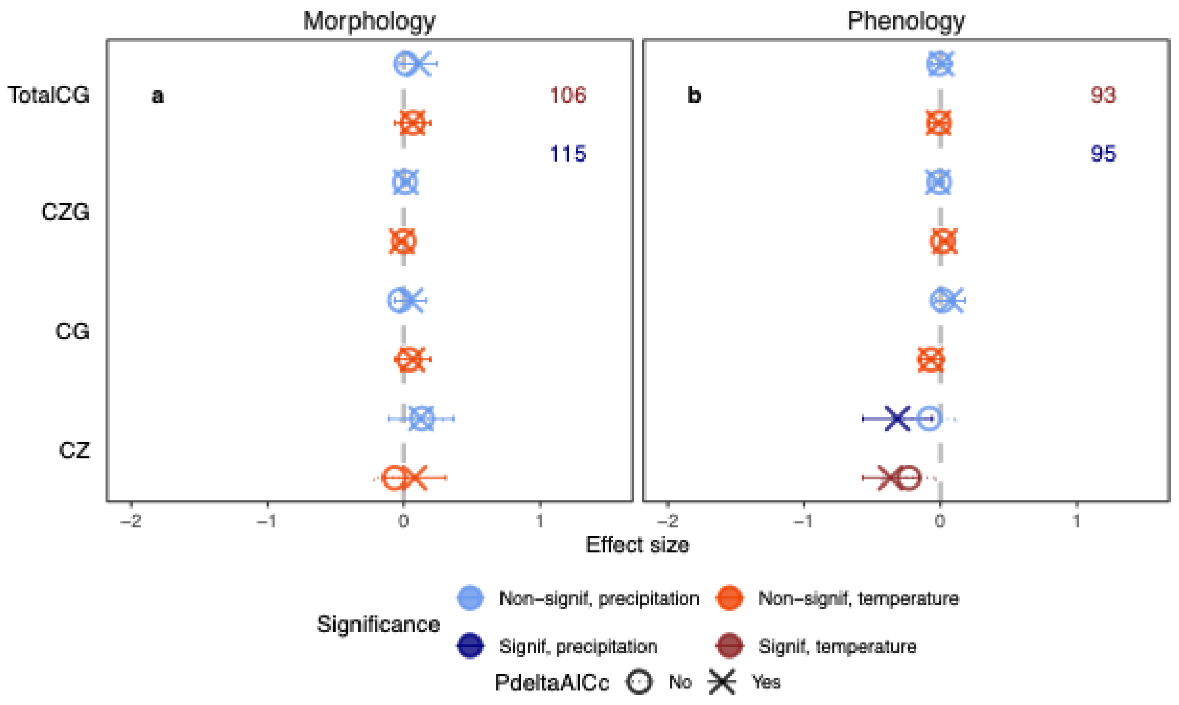

Supplementary Figure S10. The sensitivity of the meta-analyses results to the inclusion of the $P_{\Delta AICc}$ from the sliding window analysis as a covariate. Shown are four relations (CZ, CG, CZG, TotalCG), for which $P_{\Delta AICc}$ was included as a covariate in the meta-analyses. Results are shown separately for a) morphological and b) phenological traits. Significant /non-significant across-study effects (determined by the overlap of CI with 0) are shown in dark red/orange and dark blue/light blue for temperature and precipitation, respectively. The vertical grey dashed line indicates an effect size of 0. Qualitatively, the results remain unchanged if $P_{\Delta AICc}$ is not included, however, the inclusion of the $P_{\Delta AICc}$ leads to the larger absolute magnitude of the across-study estimates of the climate effects on traits (CZ) and its inclusion results in precipitation being significantly associated with phenology (CZ, compare dark blue cross vs light blue open circle in panel b) whereas this association would be non-significant otherwise. Numbers of studies for each meta-analysis are given in the upper right corner: studies focusing on temperature in red and those focusing on precipitation in blue.

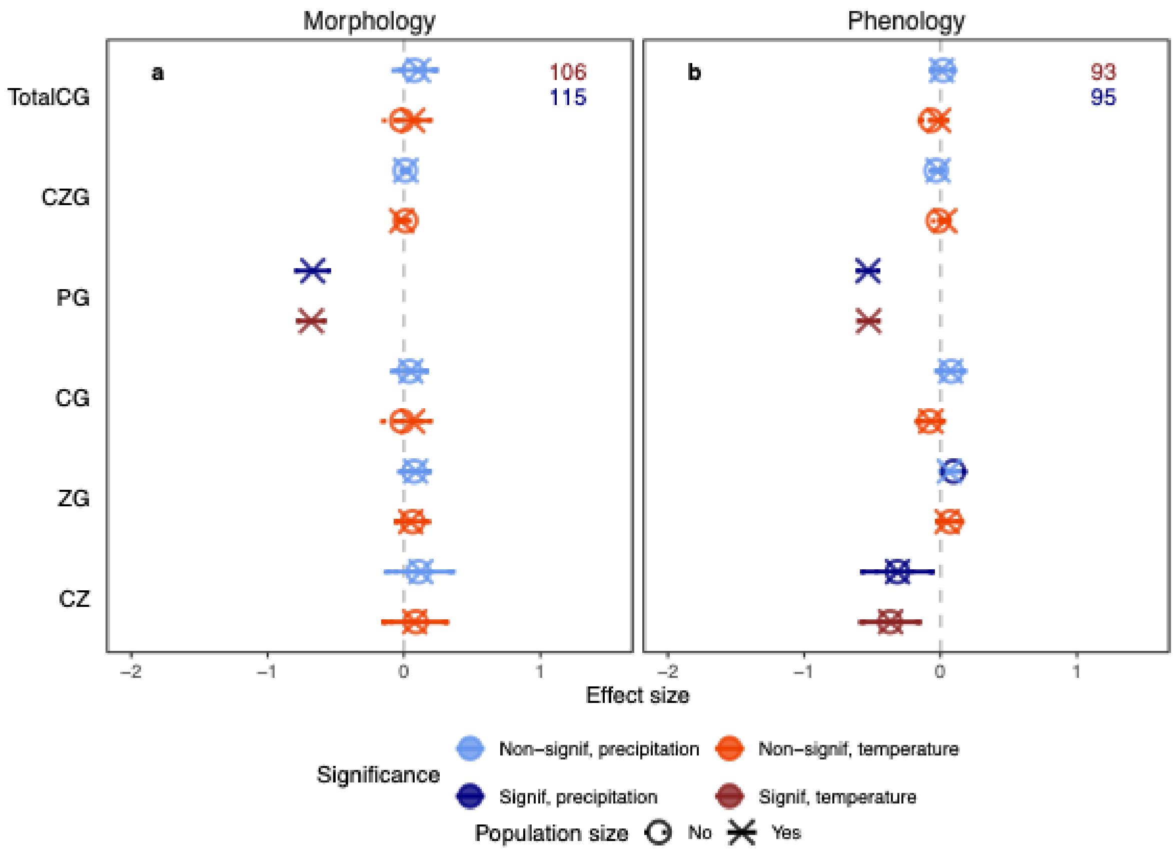

Supplementary Figure S11. Results of meta-analyses are mainly non-sensitive to the inclusion of population size in path analysis. The across-study effect sizes for each path in the path diagram (Fig. 1, main text) are shown for the analyses performed with (a cross symbol for ‘Yes’) and without (an empty circle symbol for ‘No’) the effect of population size on population growth rate (signified as DD on the figure legend). Results are shown separately for a) morphological and b) phenological traits. Significant /non-significant across-study effects (determined by the overlap of CI with 0) are shown in dark red/orange and dark blue/light blue for temperature and precipitation, respectively. The vertical grey dashed line indicates an effect size of 0. The exclusion of population size from path analysis does not affect the meta-analysis’ results qualitatively, except that for studies on phenology (b) omitting the population size (DD on the figure legend) results in one coefficient being significant: the effect of trait on population growth rate when assessing the effects of precipitation (ZG, compare dark blue circle vs light blue cross). Numbers of studies for each meta-analysis are given in the upper right corner: studies focusing on temperature in red and those focusing on precipitation in blue.

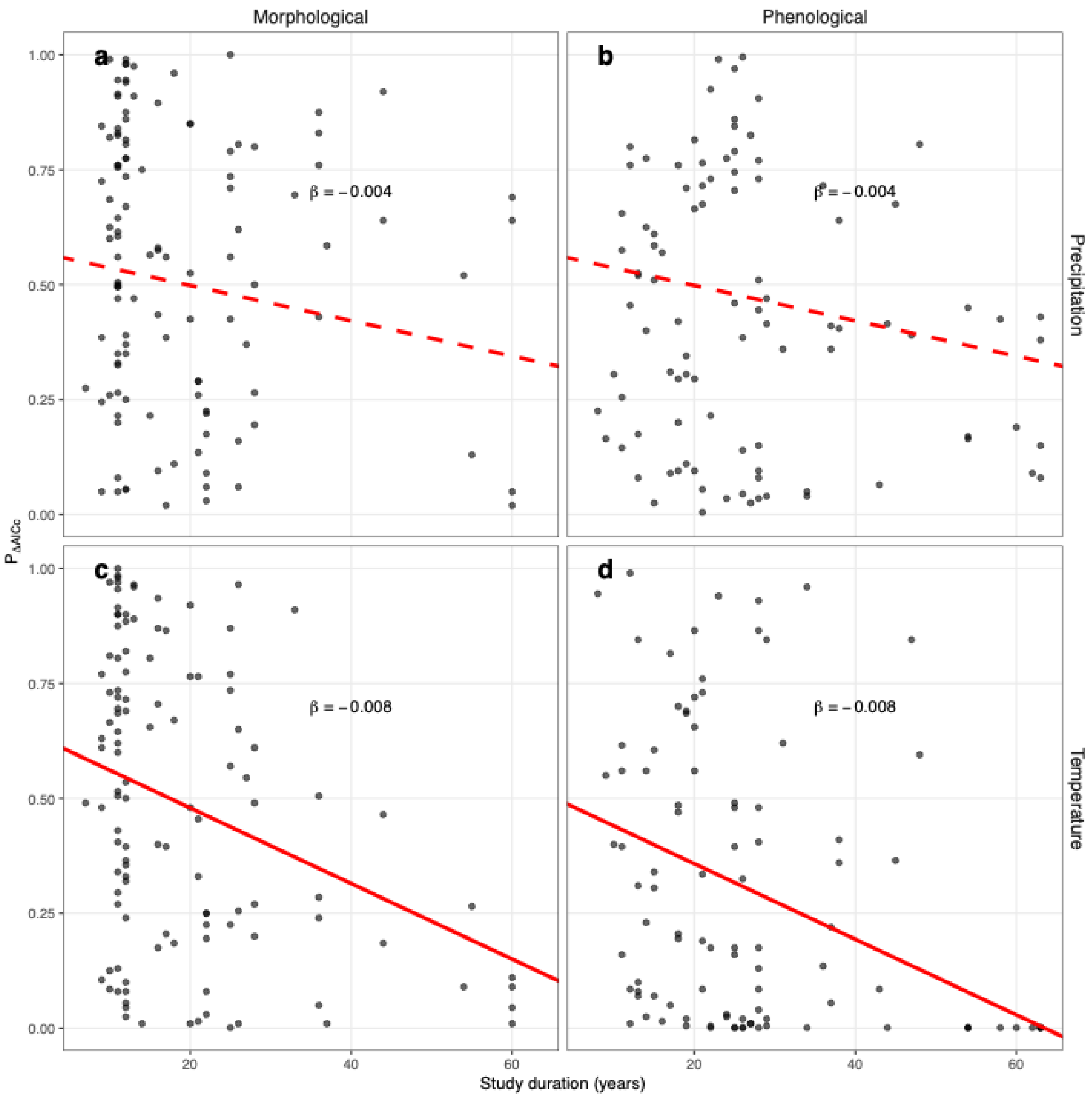

Supplementary Figure S12. The associations between study duration and the probability that the detected climate signals are spurious ($P_{\Delta AICc}$, obtained with the sliding window analyses). The probability that the detected climate signals are spurious ($P_{\Delta AICc}$) does not differ between morphological (a) and phenological traits (b) for studies on precipitation (linear model, difference between phenological and morphological traits = 0.08, df = 1, F = 3.7, p = 0.06), and was on average higher for morphological (c) compared to phenological traits (d) for studies on temperature (linear model, difference between morphological and phenological traits = 0.121, df = 1, F = 8.12, p = 0.005) and decreased with the study duration for temperature studies (linear model, β = -0.008, F = 27.7, p < 0.001). The probability that the detected climate signals are spurious ($P_{\Delta AICc}$) obtained with the sliding window analyses tended to decrease with the study duration for precipitation studies (linear model, β = -0.003, F = 3.9, p = 0.051). We fitted separate linear models (shown with red lines) to studies on precipitation and temperature, with response variable being $P_{\Delta AICc}$ and predictors trait category, study duration, and their interaction.

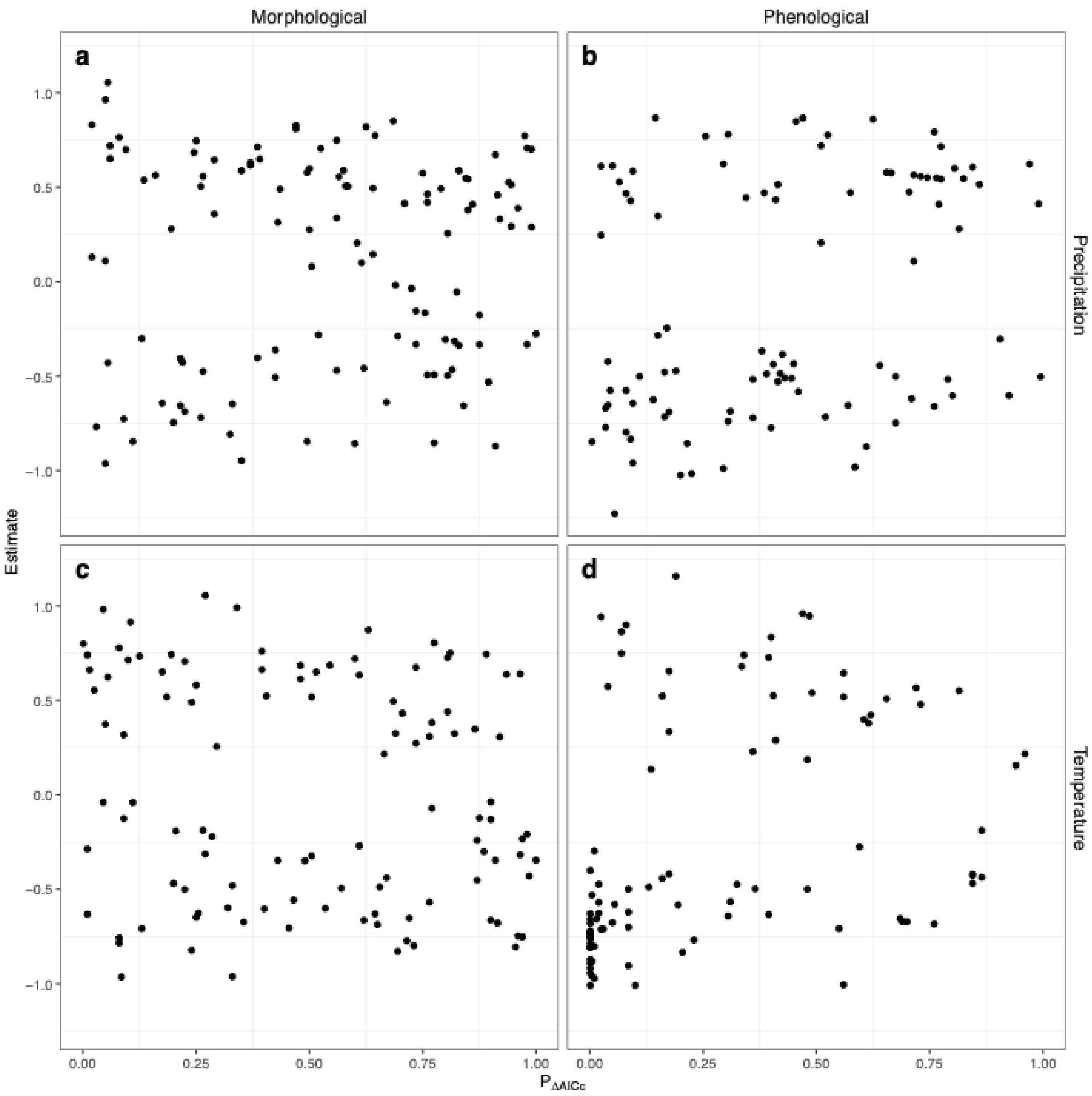

Supplementary Figure S13. Relations between the probability that the detected climate signals are spurious ($P_{\Delta AICc}$) and the path coefficient estimates for the effect of climate on trait (CZ). Results are shown separately for precipitation (a, b) and temperature (c, d) and for morphological (a, c) and phenological traits (b, d). For both trait categories, across the range of $P_{\Delta AICc}$-values we observe both positive and negative CZs (the binomial distribution that is highlighted in the main text also).

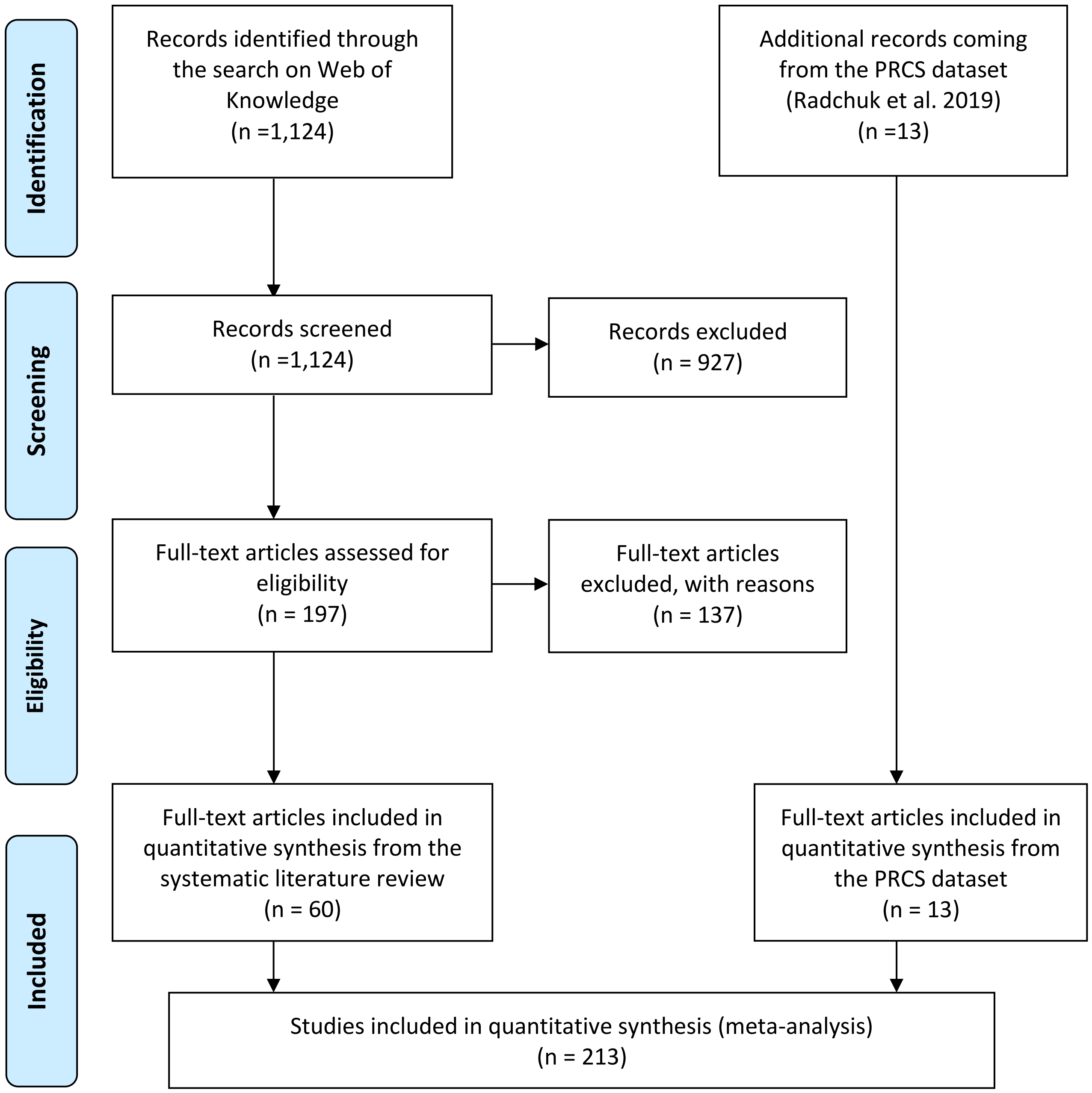

Supplementary Figure S14. A flowchart showing the number of studies included at each stage of the systematic literature review, and how these studies were combined with those coming from the PRCS dataset from (Radchuk et al., 2019). A flowchart follows the structure of the PRISMA flow diagram.

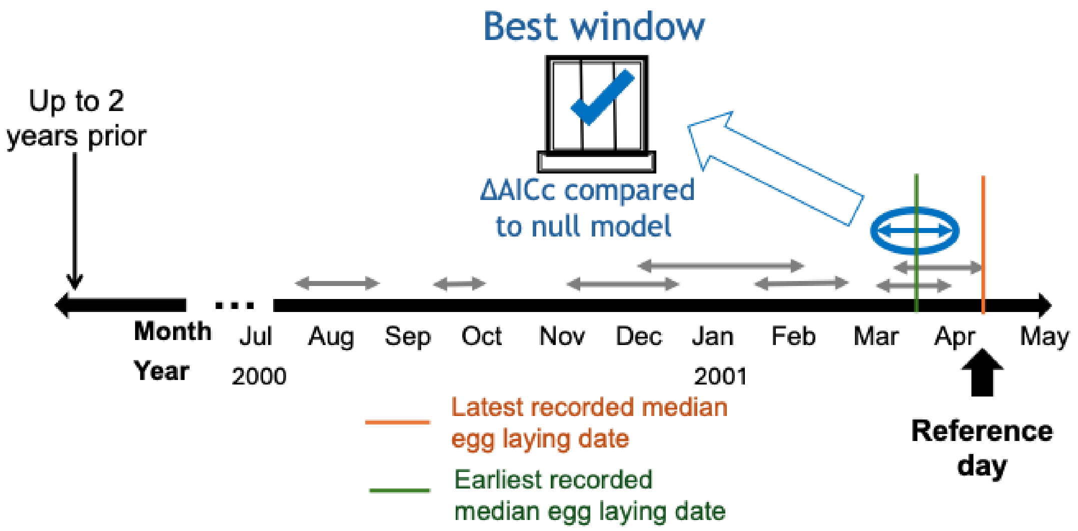

Supplementary Figure S15. Schematic representation of the sliding window analysis and the rationale for using the latest date when the phenological event was observed or morphological trait was measured as a reference day. In this hypothetic study the phenological trait (median egg laying date) of passerine birds was recorded over 10 years, from 2000 to 2009. The example focuses on the year 2001 and the orange and the green vertical lines depict, respectively, the latest and the earliest recorded median egg laying date over the 10-year study period. The grey double-headed arrows show (a selection) of tested windows. The blue double-headed horizontal arrow shows the best selected climatic window. Had we used the earliest median egg laying date recorded over the 10 years as the reference day, we would have failed to identify this climatic window (as any climatic window when using this reference day must stop at the latest time point depicted with green vertical line). Note that if the best selected window occurs after the earliest recorded egg laying date, it may not be relevant for egg laying dates observed in each year during the study (as in some years it may potentially occur after the egg laying started). By using the latest day when the event was observed as our reference day, we thus try to strike a balance between false negatives and false positives.

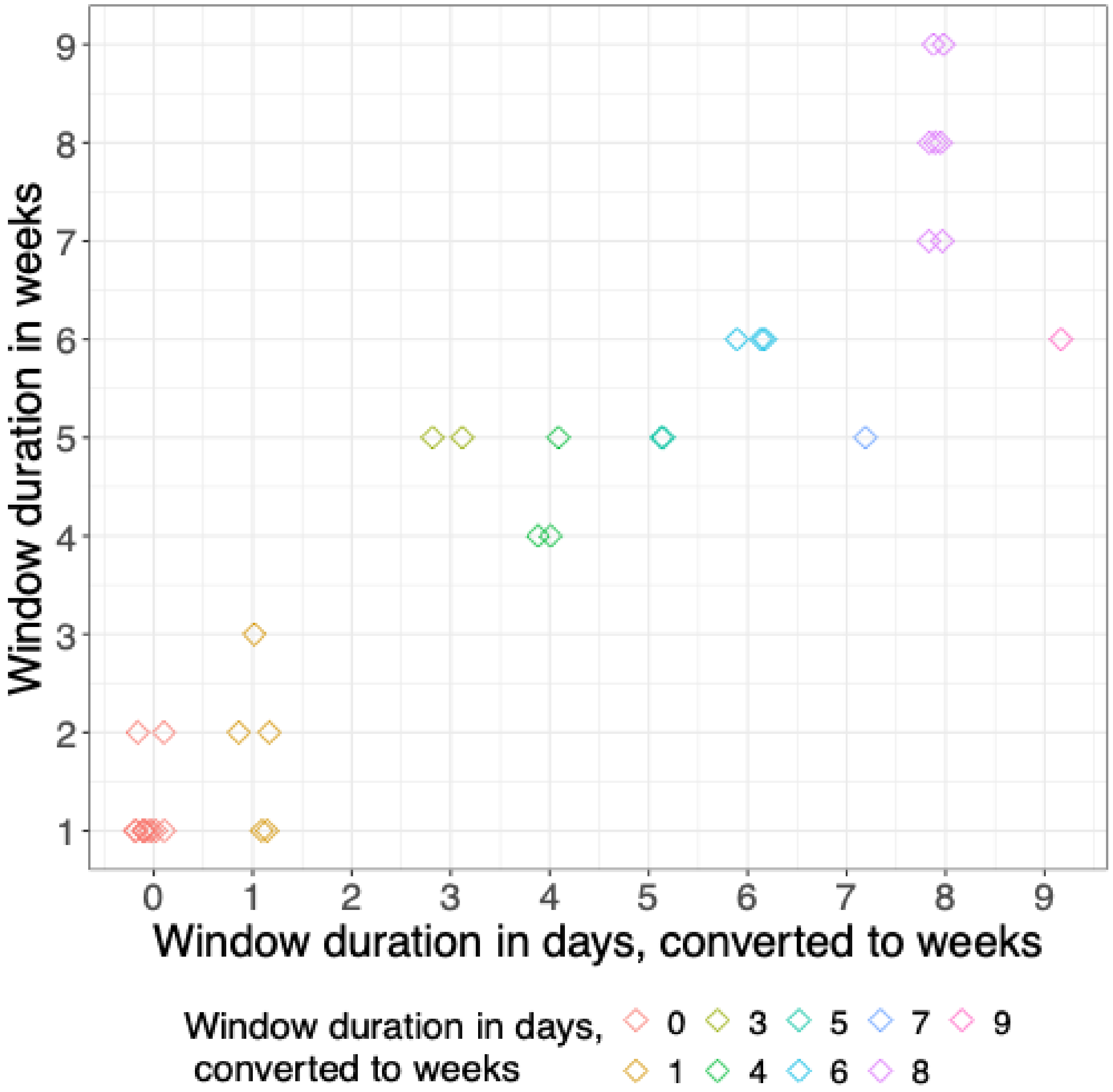

Supplementary Figure S16. The window durations calculated for a subset of 35 studies using the sliding window analysis with daily resolution (on the x axis) vs weekly resolution (on the y axis). The window duration calculated with daily resolution is converted into duration in days by dividing by 7 and rounding. The points are slightly jittered in horizontal direction to avoid overlap due to numbers being integers. The Pearson correlation between window durations computed using both resolutions is *r* (df = 33) = 0.95, p < 0.0001. We chose 35 studies as a compromise between running time and having a broad enough and representative sample of all studies. Running sliding window analysis for a study with duration of 30 years using daily resolution on a computing cluster required around one day while the same analyses with weekly resolution required two-three hours. We therefore have chosen 35 studies that constitute >1/3 of phenological studies and focus on birds only (as birds represent 87% of all studies).

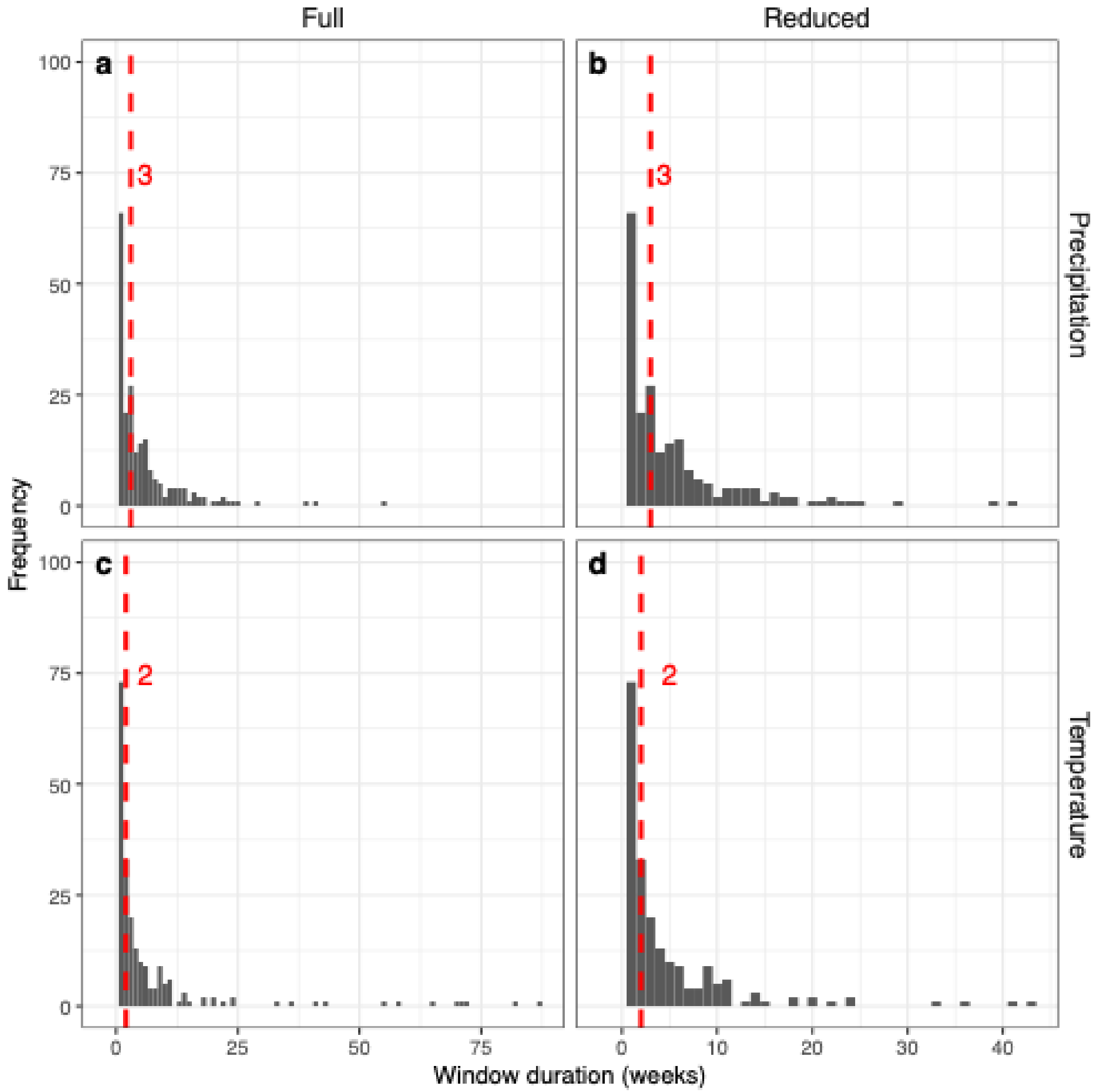

Supplementary Figure S17. Histograms of the window durations identified with the sliding window analyses performed for each climatic variable. Analyses are for (a, b) precipitation and (c, d) temperature. Panels in the left column (a and c) show the window durations of all studies, and panels in the right column (b and d) show the window durations for the subset of studies (with window duration < 52 weeks) that was used for all subsequent analyses. The vertical dashed red line and the number next to it show the median window duration across the studies. Note that the range of the x axis differs among panels.

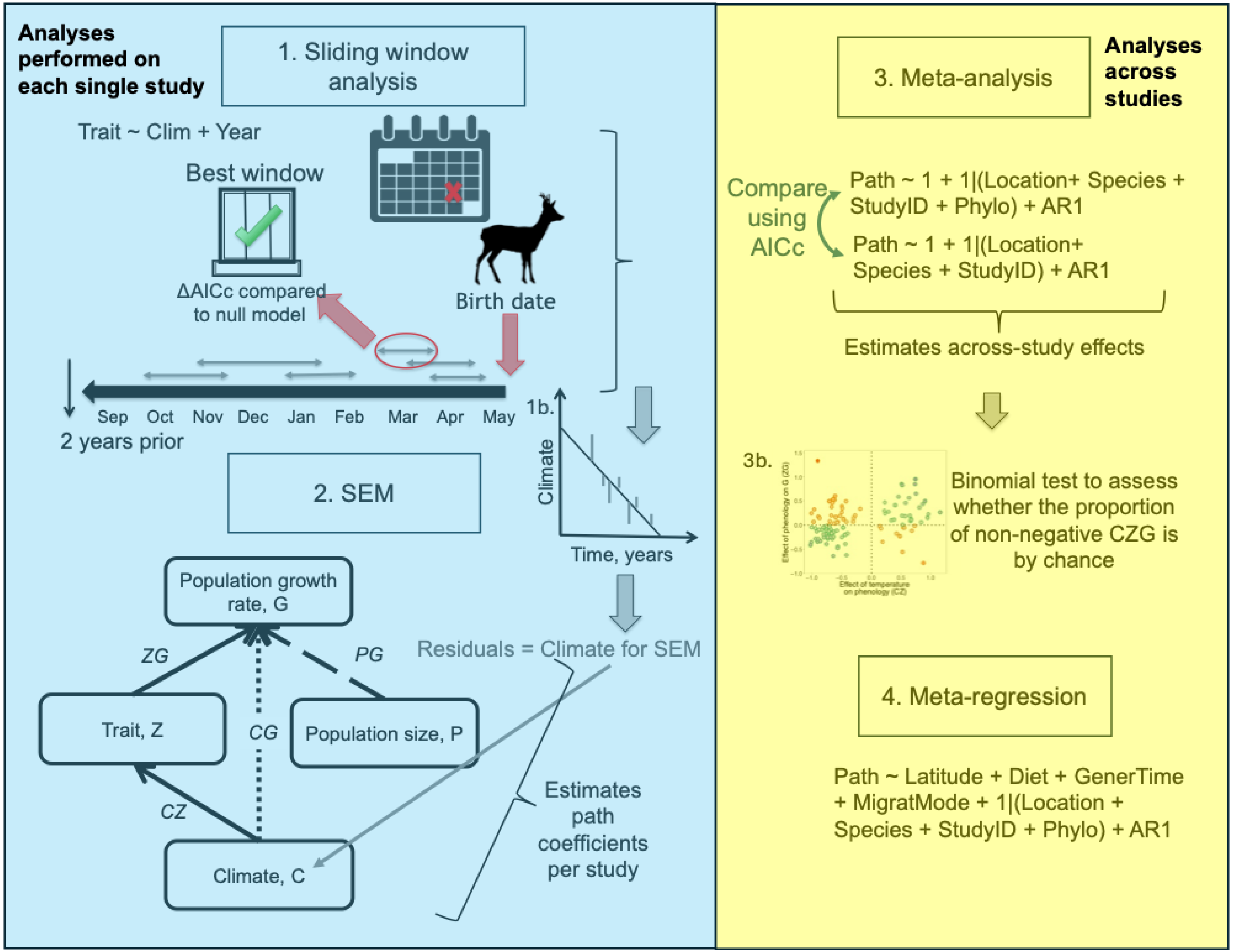
Analyses performed on each single study
1. Sliding window analysis
Trait ~ Clim + Year
Best window
ΔAICc compared to null model
Birth date
Sep Oct Nov Dec Jan Feb Mar Apr May
2 years prior
1b.
Climate
Time, years
2. SEM
Residuals = Climate for SEM
Population growth rate, G
ZG
PG
CG
Trait, Z
Population size, P
CZ
Climate, C
Estimates path coefficients per study
3. Meta-analysis
Analyses across studies
Compare using AICc
Path ~ 1 + 1|(Location+ Species + StudyID + Phylo) + AR1
Path ~ 1 + 1|(Location+ Species + StudyID) + AR1
Estimates across-study effects
3b.
Binomial test to assess whether the proportion of non-negative CZG is by chance
4. Meta-regression
Path ~ Latitude + Diet + GenerTime + MigratMode + 1|(Location + Species + StudyID + Phylo) + AR1

Supplementary Fig. S18. Schematic workflow showing the analyses performed in this study. The blue-shaded section shows the analyses performed on each single study in the dataset and the yellow-shaded section shows the analyses that were performed across all the studies. 1b) shows that we year-detrended the climate variable that was identified with the sliding window analyses prior to fitting SEMs. In other words, in SEM analyses as climate variables we used residuals from the model with climate values (obtained with sliding window analyses) as a response and time as a predictor. 3b) schematically shows that a binomial test was used to assess whether the proportion of non-negative CZG values was higher than expected by chance.

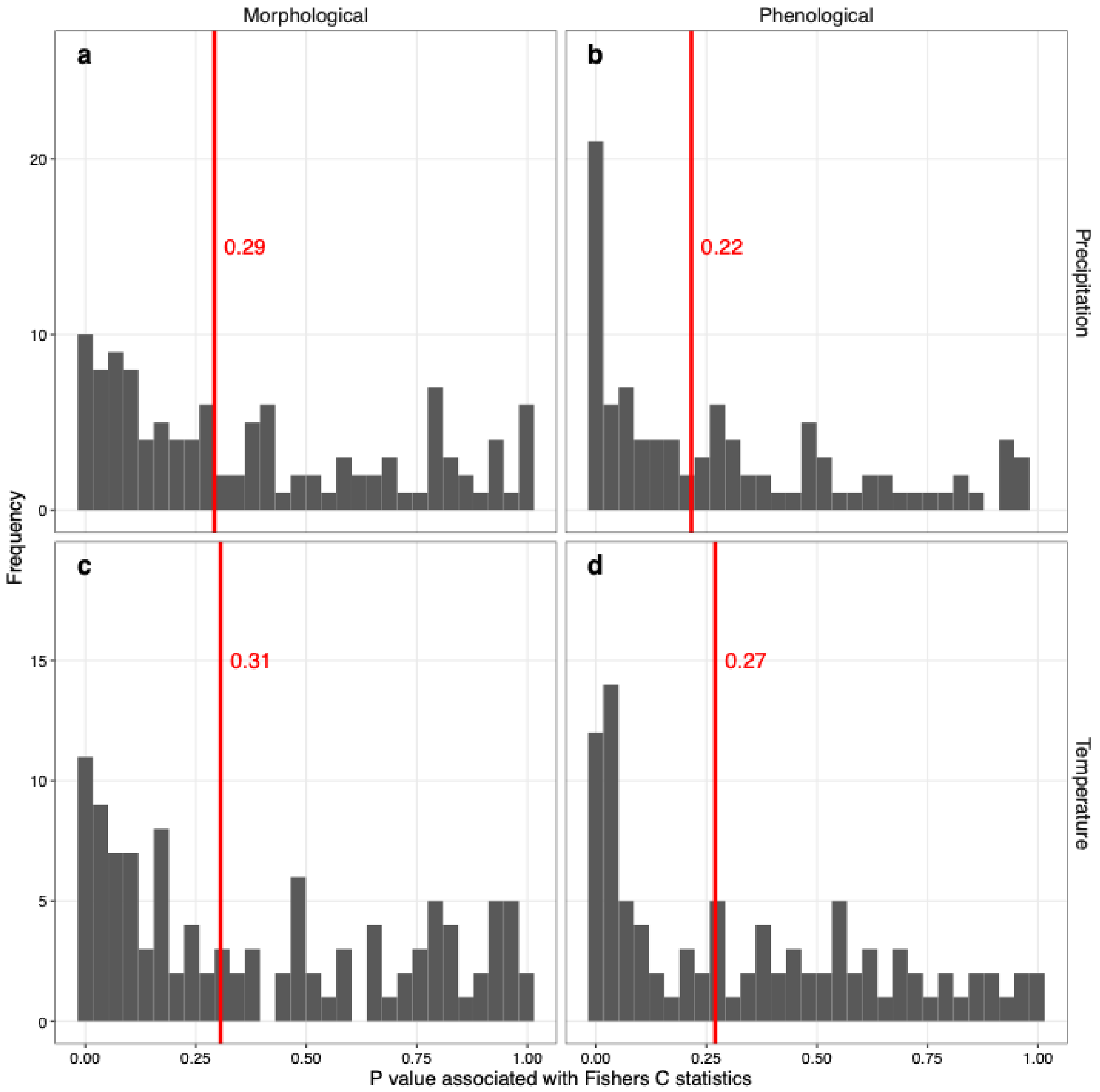

Supplementary Figure S19. Goodness of fit of the fitted SEMs. Histograms show the p-value assessing the significance of the Fisher's C statistic, separate for the studies in each trait category: morphological (a, c) and phenological traits (b, d) and for each climate variable: precipitation (a, b) and temperature (c, d). The vertical red line and the value next to it show the median p-value across the studies in each group. The goodness of fit test was satisfied for 72% of phenological studies and 84% of morphological studies (i.e. p-value > 0.05). We retained all the studies for subsequent analyses as explained in the Supplementary Methods.

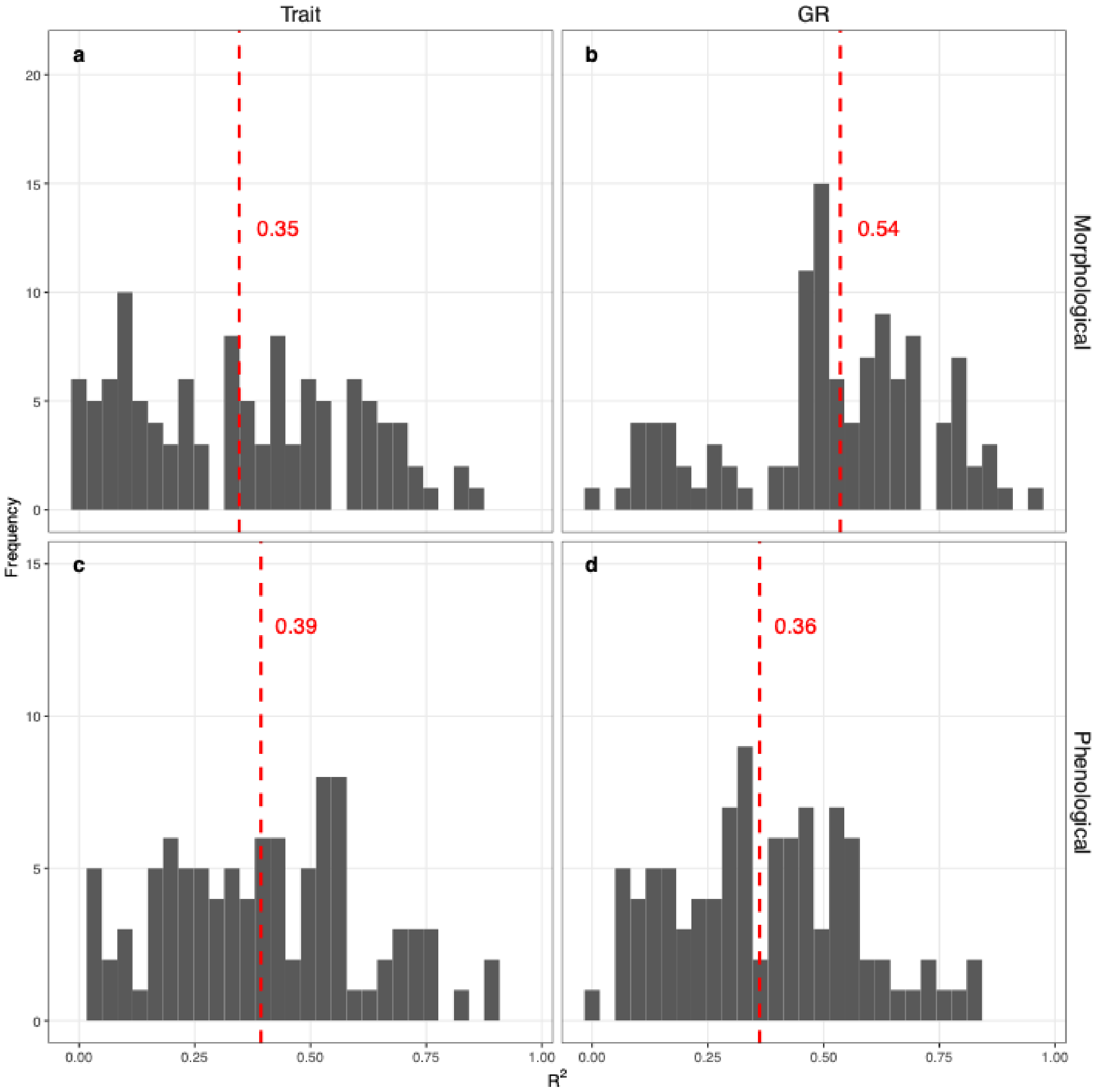

Supplementary Figure S20. R^2 of the two models constituting SEMs that were fitted to explain the effect of temperature$_d$. The response variables in these models are: the trait (a, c) and population growth rate (GR: b, d). Results are shown separately for the studies on morphological (a, b) and phenological traits (c, d). The vertical red dashed line and the value next to it show the median R^2 across the studies in each category.

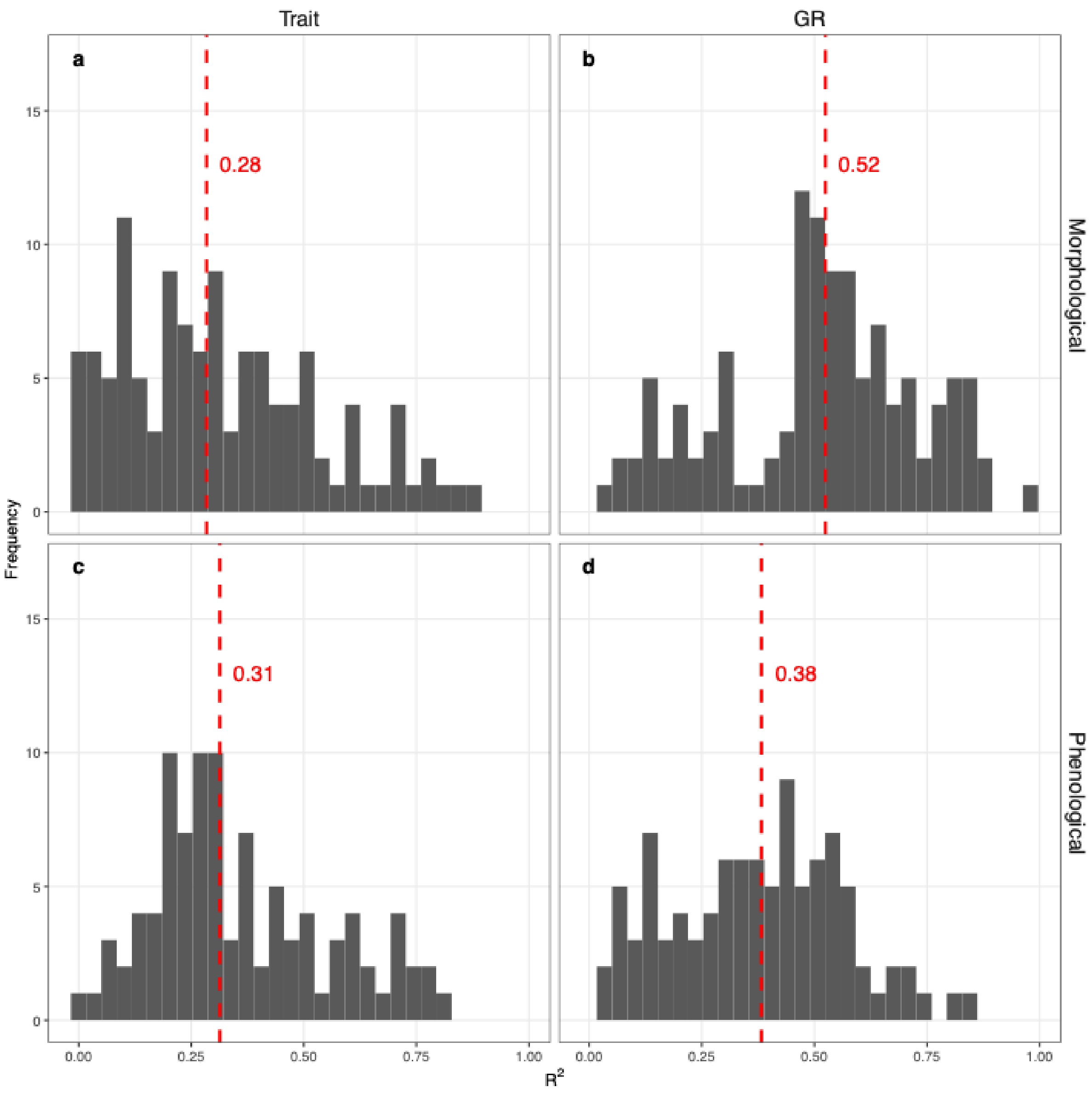

Supplementary Figure S21. R^2 of the two models constituting SEMs that were fitted to explain the effect of precipitation$_d$. The response variables in these models are: the trait (a, c) and population growth rate (GR: b, d). Results are shown separately for the studies on morphological (a, b) and phenological traits (c, d). The vertical red dashed line and the value next to it show the median R^2 across the studies in each category.

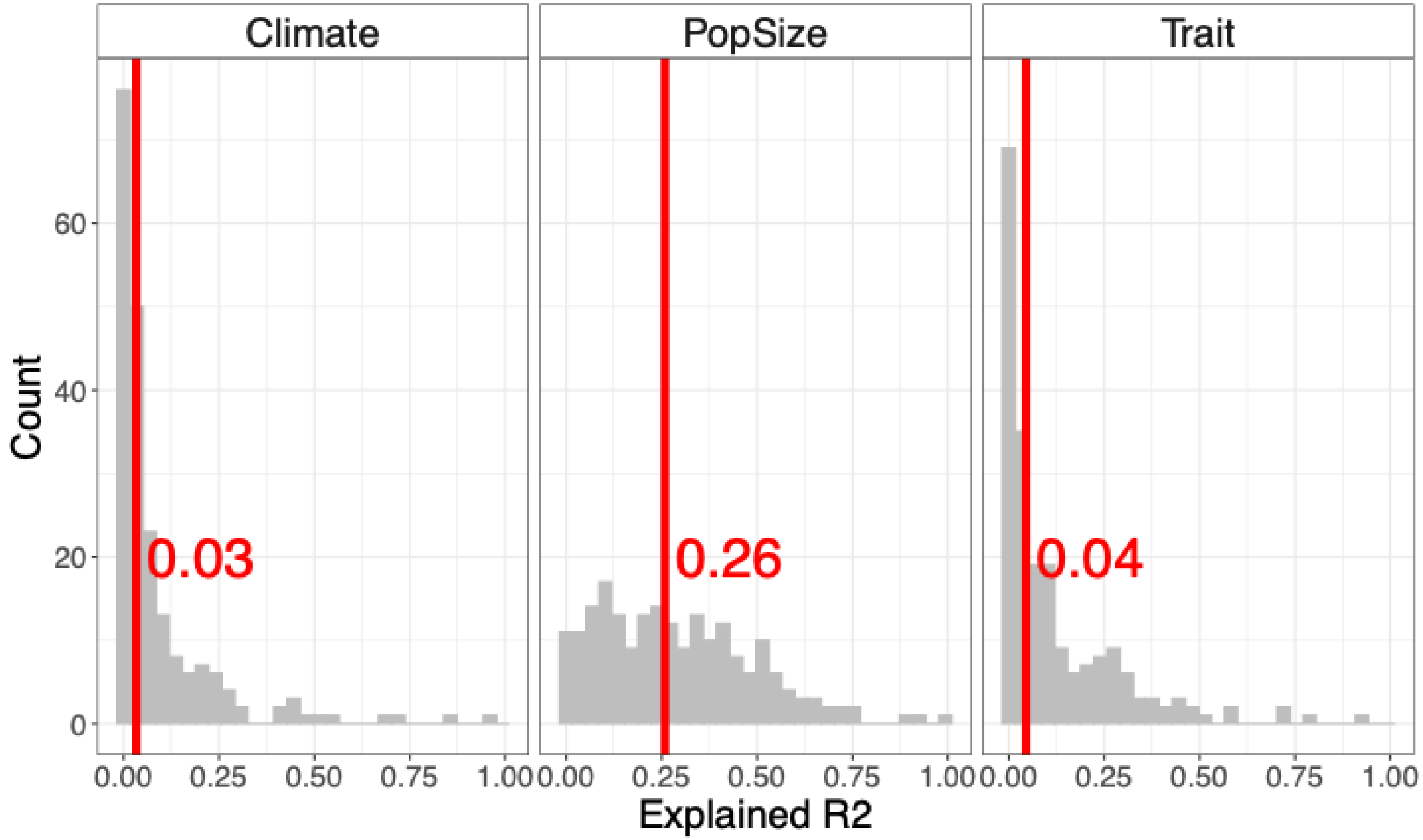

Supplementary Figure S22. Proportion of variance in G explained by temperature (“Climate”), population size (“PopSize”) and phenology (“Trait”), across all models. The variance partitioning was applied to the “population growth model” that was a part of our SEM (see Methods, “Trait-mediated effects of climate on G” and Fig. 1). The red vertical line and the text next to it shows the median across the studies.

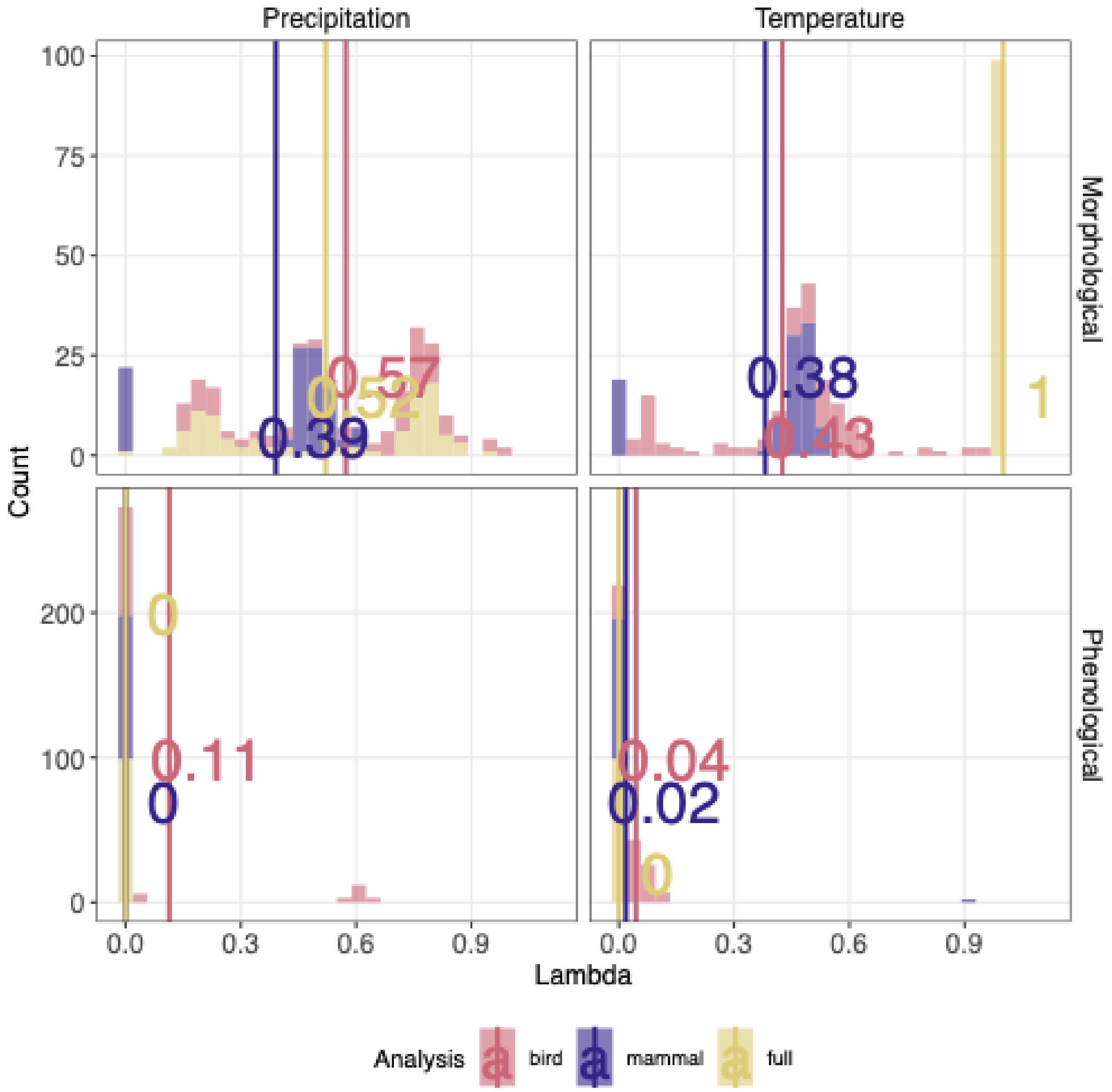

Supplementary Fig. S23. Histograms of Pagel's λs obtained across 100 models fitted to 100 random drawn posterior vertebrate mega-trees. The models are fitted to explain the effect of climate on trait (CZ, see Fig. 1 in the main text) for each combination of phenotypic traits and climate variable. Different shades show the results of the analyses run on the full dataset or separately for birds and mammals. The vertical lines and text next to them show the mean λ across all the models fitted for the randomly drawn 100 trees.

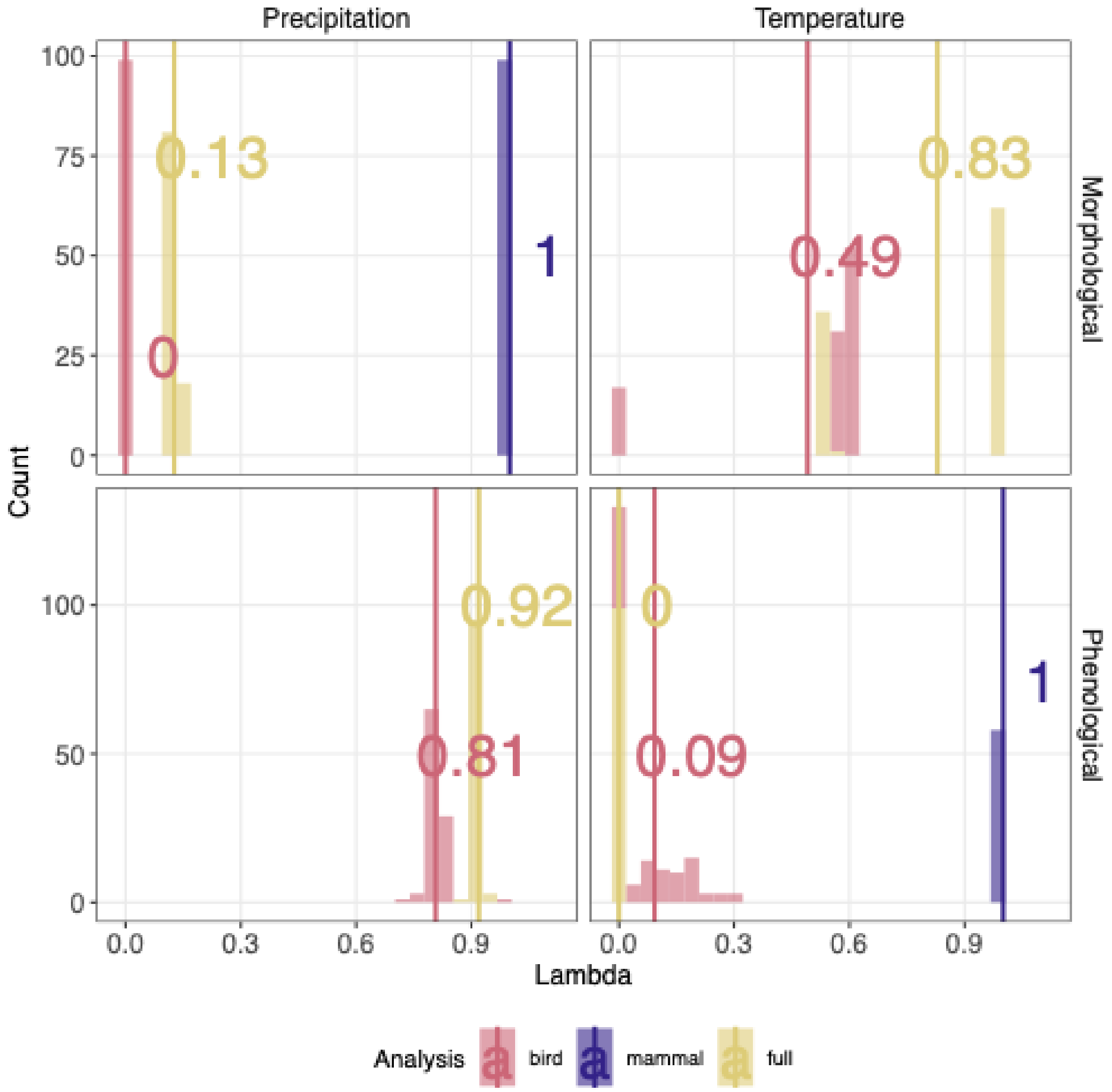

Supplementary Figure S24. Histograms of Pagel's λs obtained across 100 models fitted to 100 random drawn posterior vertebrate mega-trees. The models are fitted to explain the effect of trait on population growth rate (ZG) after accounting for both the direct effects of climate and population size (see Fig. 1 in the main text) for each combination of phenotypic traits and climate variable. Different shades show the results of the analyses run on the full dataset or separately for birds and mammals. The vertical lines and text next to them show the mean λ across all the models fitted for the randomly drawn 100 trees.

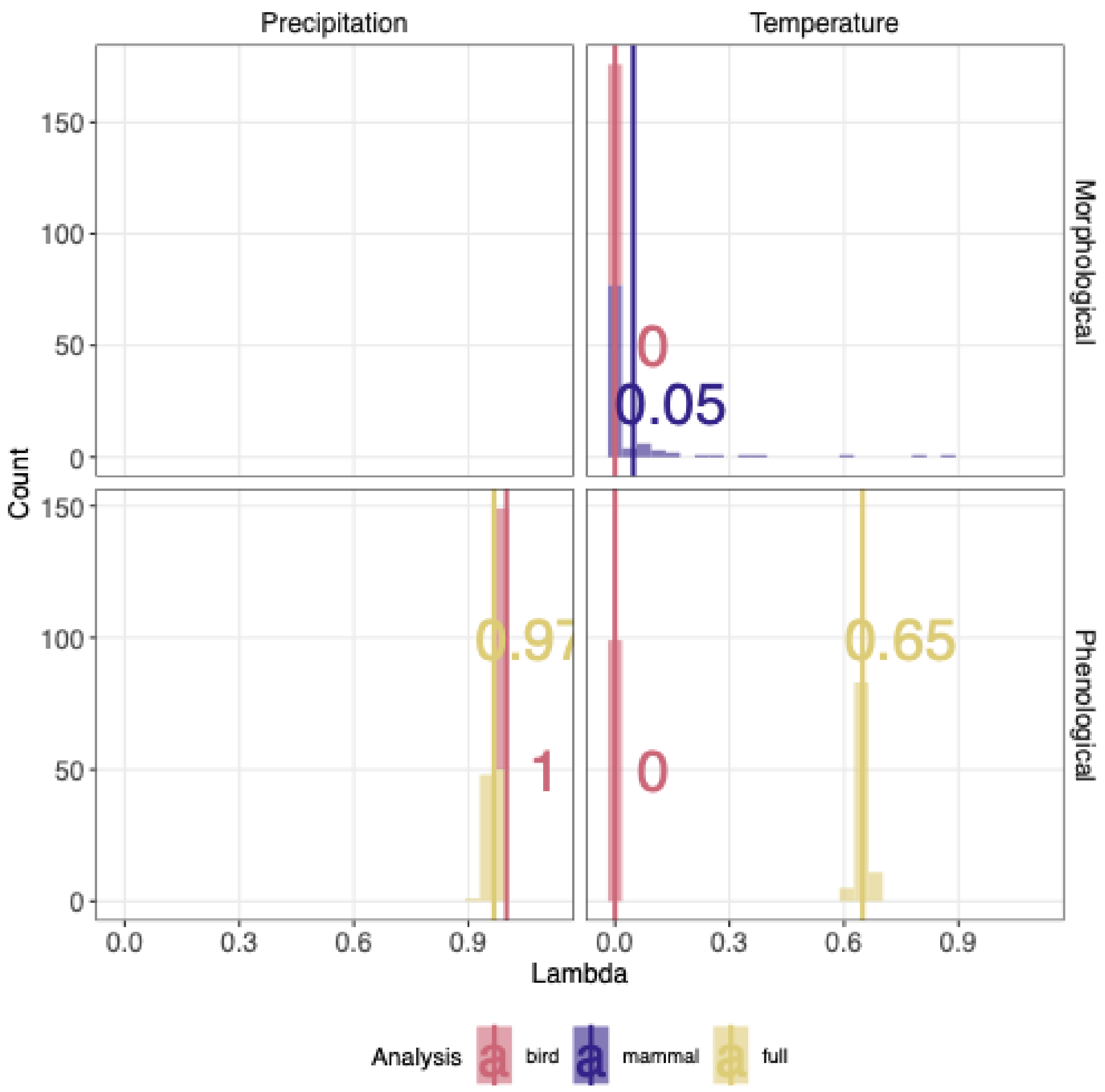

Supplementary Figure S25. Histograms of Pagel's λs obtained across 100 models fitted to 100 random drawn posterior vertebrate mega-trees. The models are fitted to explain the trait-mediated effect of climate on population growth rate (CZG, see Fig. 1 in the main text) for each combination of phenotypic traits and climate variable. Different shades show the results of the analyses run on the full dataset or separately for birds and mammals. The vertical lines and text next to them show the mean λ across all the models fitted for the randomly drawn 100 trees. For mammals in case of phenological responses to temperature Pagel's λ could not be estimated in none of the models because of too few data points (n = 7).

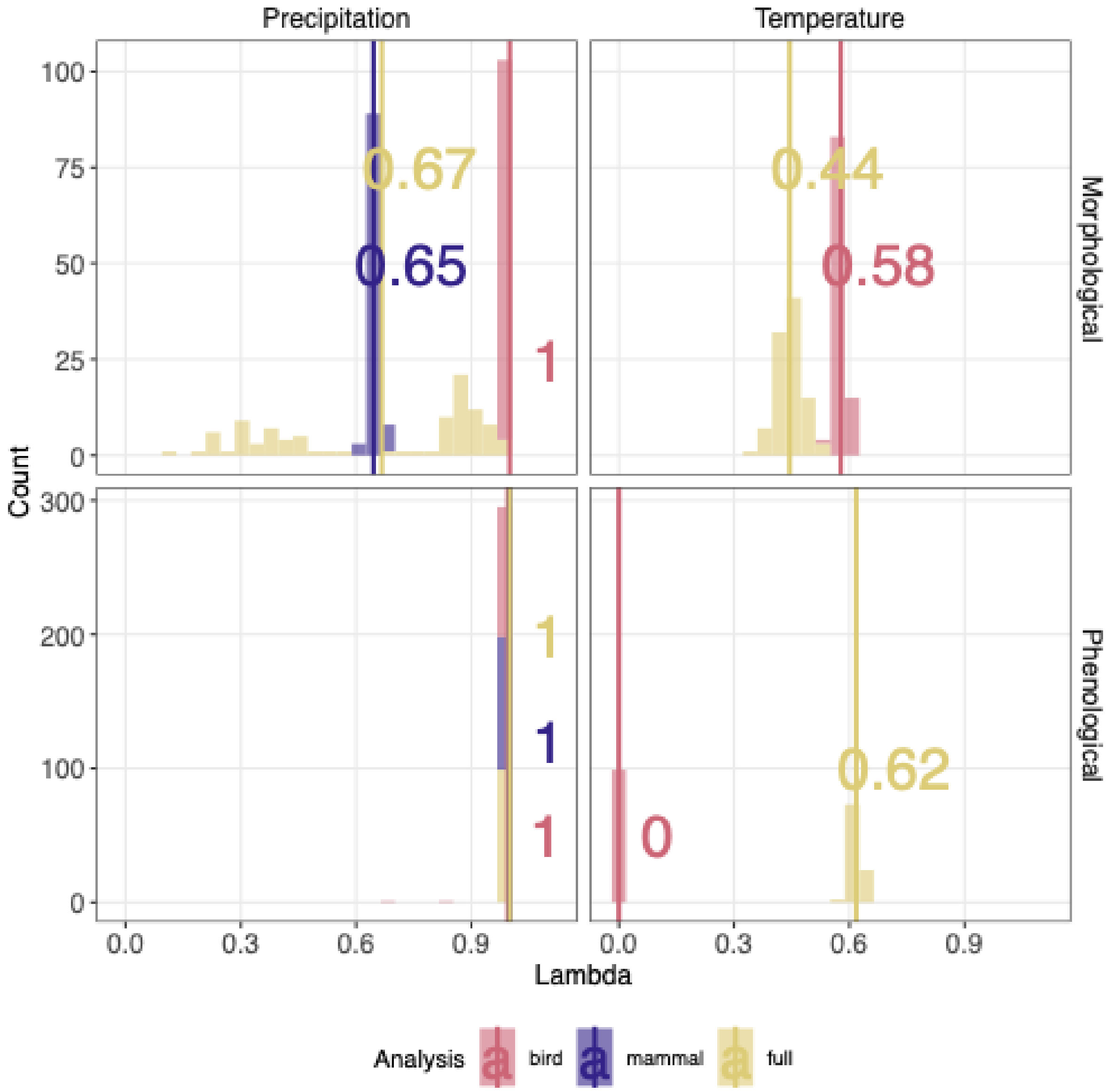

Supplementary Figure S26. Histograms of Pagel's λs obtained across 100 models fitted to 100 random drawn posterior vertebrate mega-trees. The models are fitted to explain the direct effect of climate on population growth rate, i.e. not mediated by the focal trait (CG, see Fig. 1 in the main text) for each combination of phenotypic traits and climate variable. Different shades show the results of the analyses run on the full dataset or separately for birds and mammals. The vertical lines and text next to them show the mean λ across all the models fitted for the randomly drawn 100 trees. For mammals in case of both phenological and morphological responses to temperature λ could not be estimated in any of the models because of too few data points.

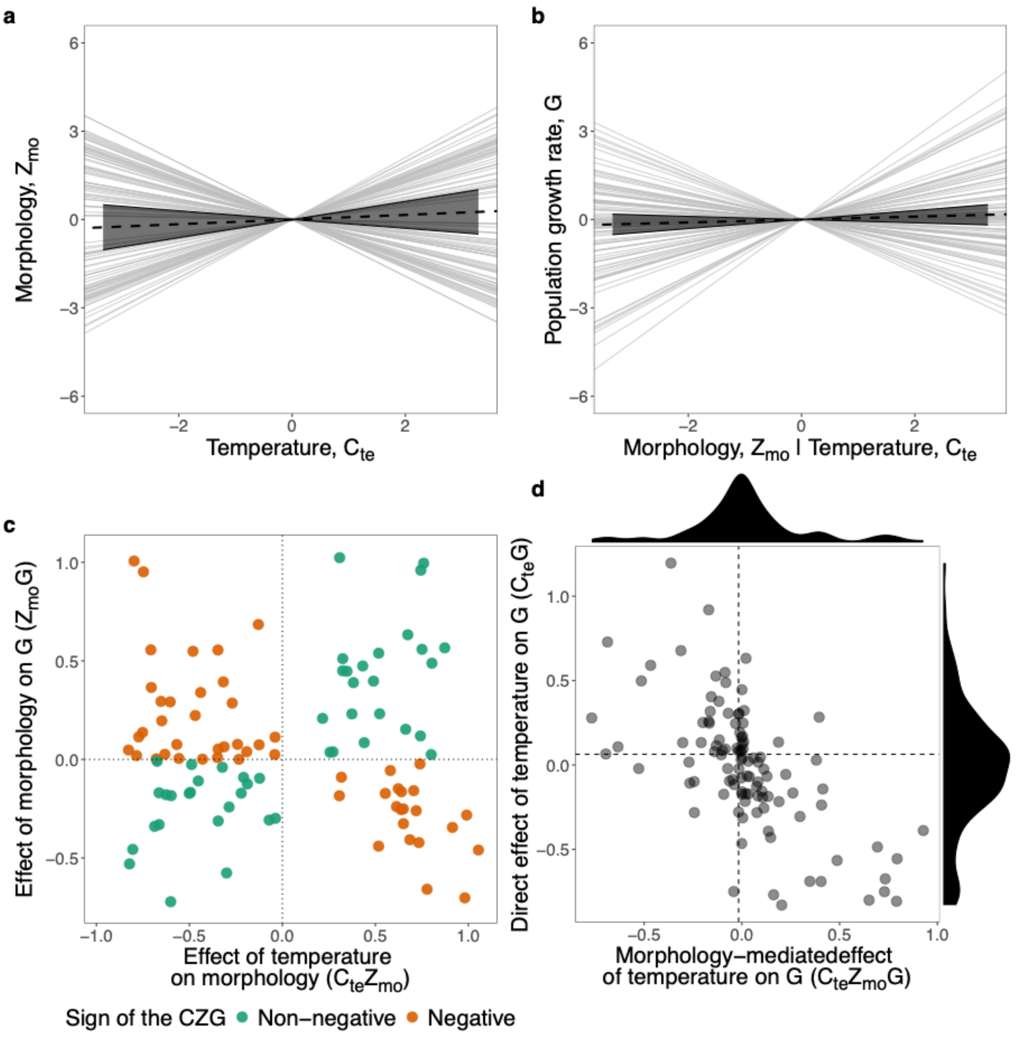

Supplementary Figure S27. Application of our conceptual framework to the associations between morphological traits and temperature. Relations between temperature$_d$ and morphological traits (a), morphological traits and population growth rate, conditional on temperature$_d$ (b), morphological responses to temperature$_d$ and morphological effects on population growth rate (c), and morphology-mediated effect of temperature on population growth rate and the direct effect of temperature$_d$ on population growth rate (d). The subscripts to C indicate the type of the climate variable ('te' for temperature and 'pr' for precipitation) and the subscripts to Z indicate the type of the trait category ('ph' for phenology and 'mo' for morphology). Grey lines in a) and b) show estimated slopes for each single study and black lines show the across-study effects (solid line for significant effects at the p-value threshold of 0.05 and dashed for non-significant). In c) studies with non-negative CZG (≥ 0) are coloured in green and those with negative CZG – in orange. On average, across the studies, temperature$_d$ positively (but not significantly) affected morphology (a), morphology was positively but non-significantly associated with population growth rate (b), and the morphology-mediated effect of temperature on population growth rate was not significantly different from 0 (d). Associations between morphology and temperature$_d$ were not significantly correlated with the associations between morphology and population growth rate, across studies (Pearson *r* (df = 104) = 0.083, p = 0.395). The proportion of studies with non-negative morphology-mediated effect of temperature on population growth rate was not significantly larger than expected by chance (mean proportion of studies with non-negative CZG = 0.48, p = 0.68).

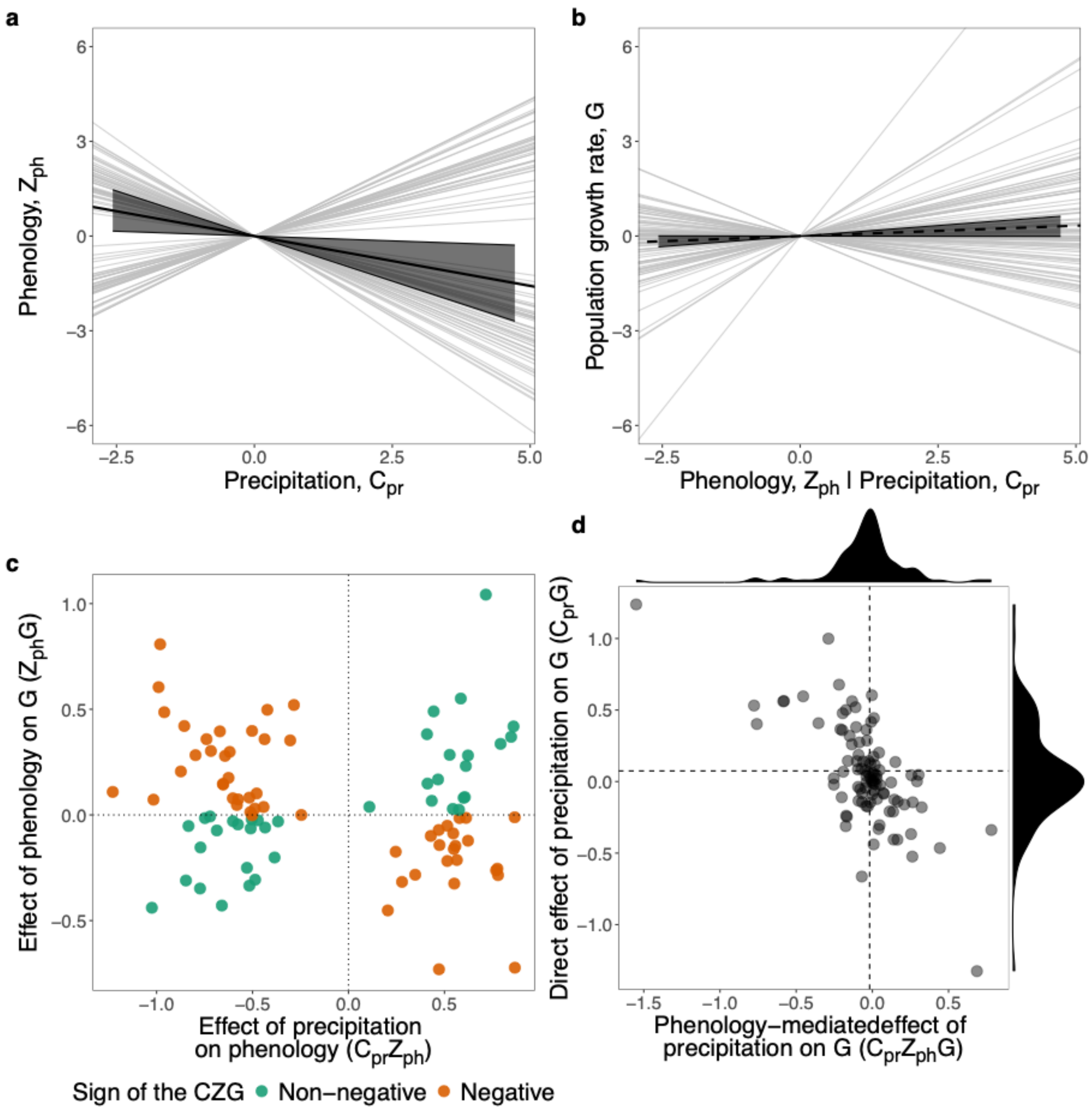

Supplementary Figure S28. Application of our conceptual framework to the associations between phenological traits and precipitation. Relations between precipitation$_d$ and phenological traits (a), phenological traits and population growth rate, conditional on precipitation$_d$ (b), phenological responses to precipitation$_d$ and phenological effects on population growth rate (c), and phenology-mediated effect of precipitation on population growth rate and the direct effect of precipitation on population growth rate (d). The subscripts to C indicate the type of climate variable (‘te’ for temperature and ‘pr’ for precipitation) and the subscripts to Z indicate the type of trait category (‘ph’ for phenology and ‘mo’ for morphology). The notations are as in Suppl. Fig. S27. On average, across the studies, precipitation$_d$ delayed phenology (a), phenology was positively but non-significantly associated with population growth rate (b), and the phenology-mediated effect of precipitation on population growth rate was not significantly different from 0 (d). Associations between phenology and precipitation$_d$ were not significantly correlated with the associations between phenology and population growth rate, across studies (Pearson r (df = 93) =-0.144, p = 0.165). The proportion of studies with non-negative phenology-mediated effect of precipitation on population growth rate was not significantly larger than expected by chance (mean proportion of studies with non-negative CZG = 0.42, p = 0.97).

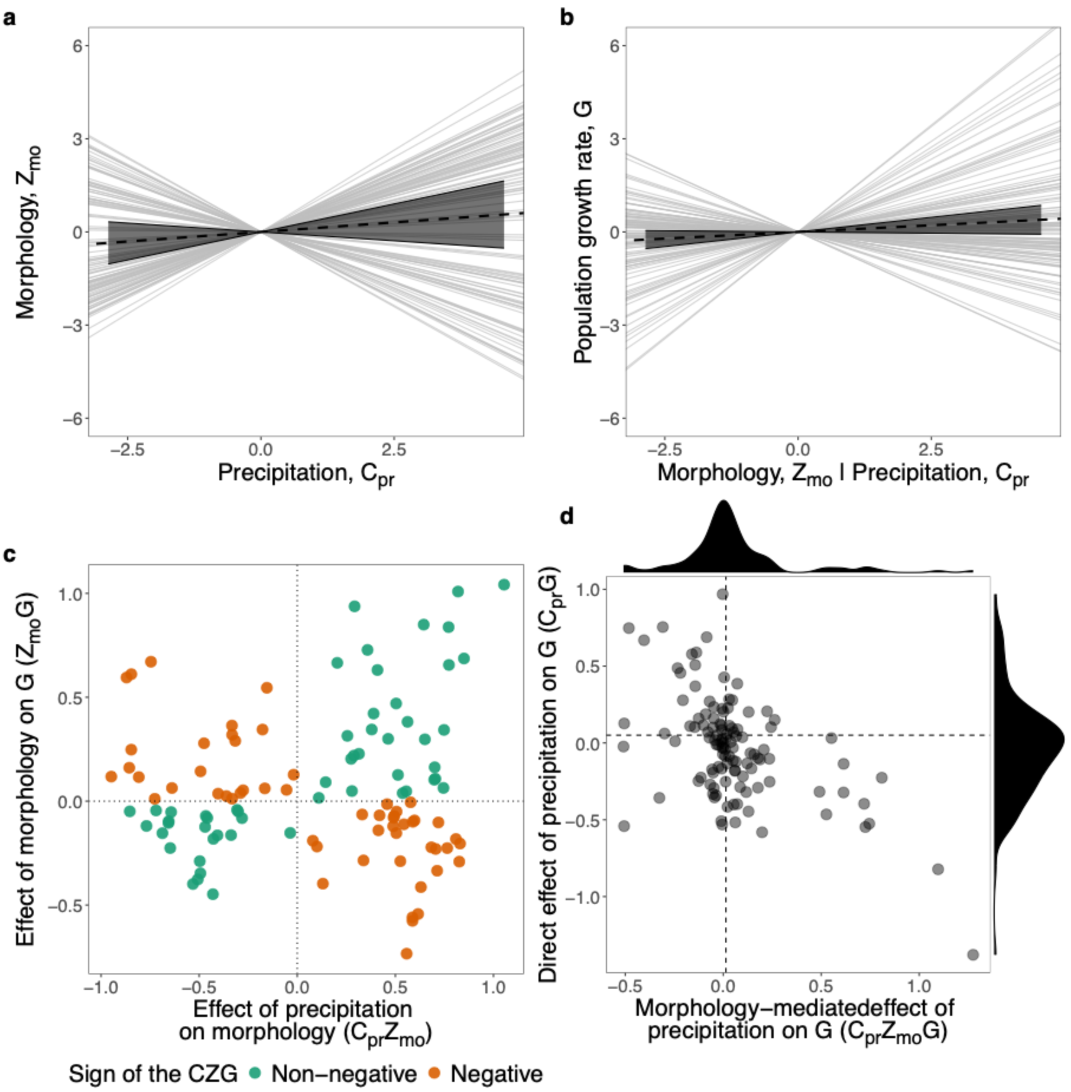

Supplementary Figure S29. Application of our conceptual framework to the associations between morphological traits and precipitation. Relations between precipitation$_d$ and morphological traits (a), morphological traits and population growth rate, conditional on precipitation (b), morphological responses to precipitation$_d$ and morphological effects on population growth rate (c), and morphology-mediated effect of precipitation on population growth rate and the direct effect of precipitation on population growth rate (d). The subscripts to C indicate the type of climate variable ('te' for temperature and 'pr' for precipitation) and the subscripts to Z indicate the type of trait category ('ph' for phenology and 'mo' for morphology). The notations are as in Suppl. Fig. S27. On average, across the studies, precipitation$_d$ was positively but non-significantly associated with morphology (a), morphology was positively but non-significantly associated with population growth rate (b), and the morphology-mediated effect of precipitation on population growth rate was not significantly different from 0 (d). Associations between morphology and precipitation$_d$ were positively but non-significantly correlated to the associations between morphology and population growth rate across the studies (Pearson *r* (df = 113)= 0.182, p = 0.0519). The proportion of studies with non-negative morphology-mediated effect of precipitation on population growth rate was not significantly larger than expected by chance (mean proportion of studies with non-negative CZG = 0.51, p = 0.43).

SUPPLEMENTARY TABLES

Supplementary Table S1. Explaining heterogeneity in phenological responses to temperature$_d$ (CZ) with the specific type of phenological trait measured. The model fitted was analogous to the meta-analytical models described in the main text but included as a predictor categorical variable "Specific phenological trait type" (and as a covariate $P_{\Delta AICc}$, the probability that the detected climatic signals were spurious obtained in the sliding window analysis). We tested significance with the two-sided Wald tests. Shown are the estimates, standard errors, lower (CI.lb) and upper (CI.ub) confidence intervals for the levels of the phenological type variable and for the $P_{\Delta AICc}$, as well as Chi2 statistic and p-values for each variable.

Variable	Level	Estimate	SE	CI.lb	CI.ub	Chi2	p-value
Specific phenological trait type	ArrivalDate	-0.237	0.247	-0.722	0.248	19.62	0.0119
	EmergenceDate	-0.775	0.406	-1.570	0.020	19.62	0.0119
	FirstLayDate	-0.396	0.304	-0.993	0.200	19.62	0.0119
	Fledging_Date	-0.333	0.625	-1.558	0.893	19.62	0.0119
	HatchingDate	-0.997	0.557	-2.089	0.095	19.62	0.0119
	OnsetBreeding	-0.290	0.110	-0.505	-0.074	19.62	0.0119
	ParturitionDate	-0.892	0.377	-1.630	-0.153	19.62	0.0119
	RutDate	-0.289	0.564	-1.395	0.816	19.62	0.0119
$P_{\Delta AICc}$	-	0.520	0.178	0.172	0.868	10.02	0.0015

Supplementary Table S2. For the analyses focusing on the effects of temperature on phenology, shown are the effects of absolute latitude and species characteristics on the path coefficient CZ (phenological response to temperature$_d$). The quantitative predictors were grand-mean-centred prior to the model fitting. Significance is estimated with the two-sided Wald test (p-values and Chi2 are shown). We used the Bonferroni correction to account for multiple comparisons, the corrected p-value threshold is 0.01 and only the intercept approached significance at this threshold (in italic). $P_{\Delta AICc}$ is the probability that the detected climatic signals were spurious, obtained with the randomization procedure in the sliding window analysis. The reference level for diet is "carnivore" and for migratory mode – "resident".

Parameter	Level	Estimate	SE	Chi2	p-value
intercept		*-0.388*	*0.152*	*-2.562*	*0.0104*
Diet	herbivore	0.439	0.237	5.446	0.0657
	omnivore	-0.157	0.201	5.446	
Migratory mode	migrant	0.203	0.162	1.571	0.2101
Generation time		0.011	0.013	0.708	0.4001
Absolute latitude		-0.015	0.006	5.568	0.0183
$P_{\Delta AICc}$		0.284	0.165	2.975	0.0846

Supplementary Table S3. Wald tests (two-sided) assessing significance of overall multiple regression mixed-effects models that test whether the trait characteristics and absolute latitude explained significant variation in each path coefficient. The models for which the overall test was significant at the p-value threshold of 0.05 are highlighted in italic.

**Path coefficient**	**df**	**Chi2**	**p-value**
CZ	*6*	*17.22*	*0.009*
ZG	6	9.13	0.104
CZG	6	6.14	0.407
CG	*6*	*21.25*	*0.002*

Supplementary Table S4. For the analyses focusing on the effects of temperature on phenology, shown are the effects of absolute latitude and species characteristics on the path coefficient CZG (effect of temperature$_d$ on population growth that is mediated by phenological traits). The quantitative predictors were grand-mean-centred prior to the model fitting. Significance is estimated with the two-sided Wald test (p-values and Chi2 are shown). We used the Bonferroni correction to account for multiple comparisons, the corrected p-value threshold is 0.01 and the effects significant at this threshold are highlighted in italic. $P_{\Delta AICc}$ is the probability that the detected climatic signals were spurious, obtained with the randomization procedure in the sliding window analysis. The reference level for diet is “carnivore” and for migratory mode – “resident”.

**Parameter**	**Estimate**	**SE**	**Chi2**	**p-value**
intercept	0.013	0.029	0.442	0.6582
Diet (herbivore)	-0.007	0.024	1.765	0.4138
Diet (omnivore)	0.040	0.035	1.765	0.4138
Migratory mode (migrant)	0.012	0.031	0.151	0.6973
Generation time	0.001	0.002	0.323	0.5698
Absolute latitude	-0.002	0.001	1.142	0.2851
$P_{\Delta AICc}$	-0.044	0.042	1.100	0.2943

Supplementary Table S5. For the analyses focusing on the effects of temperature on phenology, shown are the effects of absolute latitude and species characteristics on the path coefficient CG (the effect of temperature$_d$ on population growth rate via all other traits than phenology). The quantitative predictors were grand-mean-centred prior to model fitting. Significance is estimated with the two-sided Wald test (p-values and Chi2 are shown). We used the Bonferroni correction to account for multiple comparisons, the corrected p-value threshold is 0.01 and the effects significant at this threshold are highlighted in italic. $P_{\Delta AICc}$ is the probability that the detected climatic signals were spurious, obtained with the randomization procedure in the sliding window analysis. The reference level for diet is “carnivore” and for migratory mode – “resident”.

**Parameter**	**Level**	**Estimate**	**SE**	**Chi2**	**p-value**
intercept		-0.031	0.048	-0.648	0.5170
Diet	herbivore	0.031	0.040	1.039	0.5949
	omnivore	-0.014	0.060	1.039	0.5949
Migratory mode	migrant	-0.075	0.049	2.313	0.1283
Generation time		0.004	0.004	0.731	0.3927
Absolute latitude		*0.008*	*0.002*	*14.257*	*0.0002*
$P_{\Delta AICc}$		0.051	0.077	0.441	0.5066

Supplementary Table S6. The mixed-effect meta-analytical models fitted to explain each path coefficient (column 'Relation') per each climate variable (temperature vs precipitation), for each trait category (morphological vs phenological). For each model the variance of random effects is shown: species, study ID and location.

Climate	Trait category	Relation	Random Var due to Species	Random Var due Study ID	Random Var due to Location
Temperature	Morphology	CZ	0.0000	0.3108	0.0020
Temperature	Morphology	ZG	0.0197	0.0299	0.0260
Temperature	Morphology	CG	0.0000	0.0427	0.0073
Temperature	Morphology	PG	0.0045	0.0282	0.0494
Temperature	Morphology	CZG	0.0000	0.0000	0.0000
Temperature	Morphology	TotalCG	0.0072	0.0090	0.0059
Temperature	Phenology	CZ	0.1423	0.0154	0.1627
Temperature	Phenology	ZG	0.0120	0.0000	0.0196
Temperature	Phenology	CG	0.0121	0.0000	0.0079
Temperature	Phenology	PG	0.0116	0.0489	0.0000
Temperature	Phenology	CZG	0.0007	0.0000	0.0000
Temperature	Phenology	TotalCG	0.0000	0.0000	0.0000
Precipitation	Morphology	CZ	0.0000	0.2399	0.0580
Precipitation	Morphology	ZG	0.0199	0.0127	0.0380
Precipitation	Morphology	CG	0.0000	0.0293	0.0000
Precipitation	Morphology	PG	0.0166	0.0000	0.0963
Precipitation	Morphology	CZG	0.0000	0.0000	0.0000
Precipitation	Morphology	TotalCG	0.0104	0.0162	0.0000
Precipitation	Phenology	CZ	0.1357	0.2077	0.0178
Precipitation	Phenology	ZG	0.0038	0.0121	0.0159
Precipitation	Phenology	CG	0.0000	0.0000	0.0193
Precipitation	Phenology	PG	0.0148	0.0484	0.0000
Precipitation	Phenology	CZG	0.0000	0.0000	0.0005
Precipitation	Phenology	TotalCG	0.0000	0.0000	0.0000

Supplementary Table S7. Sources of daily climate data (for precipitation and temperature) per continent, their spatial resolution and websites from which they were taken. For seabirds instead of areal temperature we used sea surface temperature (SST), with a spatial resolution of 0.25x0.25 from the NCAR Climate Data Guide (https://climatedataguide.ucar.edu/): 'Blended Analysis of Daily SST and Ice, OISSTv2.'

**Continent**	**Climate variable**	**Source**	**Website**	**Resolution**
Europe	Precipitation Temperature	European Climate Assessment and Dataset	https://www.ecad.eu/	0.1x0.1 deg
Australia	Precipitation Temperature	AusCover	https://www.tern.org.au/	0.05x0.05 deg
North America	Precipitation	NOAA Physical Sciences Laboratory (PSL)	https://psl.noaa.gov/	0.25x0.25 deg
North America + other locations outside of Europe and Australia	Temperature	CPC 0.50x0.50 Global Daily Temperature from NOAA PSL	https://psl.noaa.gov/	0.5x0.5 deg
Locations outside of Europe, Australia or North America	Precipitation	CPC 0.50x0.50 Global Daily Unified Gauge-Based Analysis of Precipitation from NOAA PSL	https://psl.noaa.gov/	0.5x0.5 deg

Supplementary Table S8. An overview of the meta-analytical models fitted in this study, split per type of phenotypic trait category and climatic variable and by our research question. There are two main research questions, as explained in the introduction (see main text): to (i) assess how general trait-mediated effects are across the studies and to (ii) identify which type of species and regions exhibit the strongest trait-mediated effects of climate on population growth rate (using migratory mode, diet and generation time as explanatory species characteristics, and latitude to explain geographic variation among locations). To answer research question (i) we compared the model that accounted for phylogenetic relatedness to the model without such correction, and have chosen the best model using AIC, to avoid model overfitting. Models accounting for phylogeny were run 100 times, every time using a randomly selected phylogenetic tree, to account for the fact that available phylogenies are not fully resolved.

	**Phenotypic trait category**	
**Climatic variable**	Phenological	Morphological
Temperature	Research question (i):[1] CZ ~ $P_{\Delta AICc}$ + ClimQ + 1(Location + Species + Study + Phylo) + AR1<br>[2] ZG ~ 1 + 1(Location + Species + Study + Phylo) + AR1<br>[3] PG ~ 1 + 1(Location + Species + Study + Phylo) + AR1<br>[4] CZG ~ $P_{\Delta AICc}$ + ClimQ + 1(Location + Species + Study + Phylo) + AR1<br>[5] CG ~ $P_{\Delta AICc}$ + ClimQ + 1(Location + Species + Study + Phylo) + AR1<br>[6] TotalCG ~ $P_{\Delta AICc}$ + ClimQ + 1(Location + Species + Study + Phylo) + AR1	Same list of models as for phenology-temperature combination
	Research question (ii)<br>[1] CZ ~ Latitude + Diet + GenerTime + MigratMode + $P_{\Delta AICc}$ + 1(Location + Species + Study + Phylo) + AR1<br>[2] ZG ~ Latitude + Diet + GenerTime + MigratMode + 1(Location + Species + Study + Phylo) + AR1<br>[3] CZG ~ Latitude + Diet + GenerTime + MigratMode + $P_{\Delta AICc}$ + 1(Location + Species + Study + Phylo) + AR1<br>[4] CG ~ Latitude + Diet + GenerTime + MigratMode + $P_{\Delta AICc}$ + 1(Location + Species + Study + Phylo) + AR1	Not fitted because likelihood of spurious climatic windows is high according to sliding window analyses
	Auxiliary analysis to explain across-study heterogeneity:<br>CZ ~ TraitType + $P_{\Delta AICc}$ + 1(Location + Species + Study + Phylo) + AR1	

Precipitation	Same list of models as for phenology-temperature combination	Same list of models as for phenology-temperature combination
	Not fitted because likelihood of spurious climatic windows is high according to sliding window analyses	Not fitted because likelihood of spurious climatic windows is high according to sliding window analyses

Notations: CZ is the effect of climate variable C on phenotypic trait Z; ZG is the effect of phenotypic trait Z on population growth G after accounting for both the direct effects of temperature and population size; CZG is the trait-mediated effect of climate on population growth rate; CG is the effect of climate on population growth rate that is not mediated by the phenotypic trait of interest (but may be potentially mediated by other, not considered traits); PG is the effect of population size on population growth rate after accounting for both trait-mediated (CZG) and direct effect of climate (CG); TotalCG is a combined effect of climate on phenotypic trait that includes both the trait-mediated and direct effects of climate; $P_{\Delta AICc}$ is the probability of the climatic window detected with the sliding window analysis being spurious; ClimQ is the categorical climate data quality variable indicating whether the climate data were exact or approximate, i.e. taken from the closest grid located on the mainland; Latitude stands for latitude; Diet is a categorical variable for the diet type, as detailed in methods; GenerTime is generation time in years; MigratMode is a categorical variable for migratory mode; TraitType is a specific type of phenological trait recorded (e.g. arrival date, fledging date, emergence date); AR1 stands for autoregressive autocorrelation structure of first order that was applied to residuals.

Supplementary Table S9. Assessing the significance of the quadratic effect of climate on trait (column "Response" is "trait") and of trait on population growth rate (column "Response" is "Growth rate") for each combination of the climate variable (specified in column "Climate") and trait category (specified in column "Trait category"). The results are shown for analyses that included the study as random intercept or as random slope (specified in column "Random structure", for more details see Supplementary Methods). Shown are the results of the likelihood ratio tests (Chi2 and p-value).

Random structure	Response	Climate	Trait category	Chi2	p-value
Study as random intercept	Trait	Temperature	Phenology	4.76	0.029
	Trait	Temperature	Morphology	4.86	0.028
	Trait	Precipitation	Phenology	0.85	0.36
	Trait	Precipitation	Morphology	1.91	0.17
Study as random slope	Trait	Temperature	Phenology	0.176	1
	Trait	Temperature	Morphology	0.922	0.34
	Trait	Precipitation	Phenology	0.53	0.47
	Trait	Precipitation	Morphology	0.023	0.88
Study as random intercept	Growth rate	Temperature	Phenology	0.53	0.47
	Growth rate	Temperature	Morphology	1.87	0.17
	Growth rate	Precipitation	Phenology	0.56	0.45
	Growth rate	Precipitation	Morphology	1.87	0.17
Study as random slope	Growth rate	Temperature	Phenology	0.26	0.61
	Growth rate	Temperature	Morphology	1.94	0.16
	Growth rate	Precipitation	Phenology	0.284	0.59
	Growth rate	Precipitation	Morphology	1.937	0.164

Supplementary Table S10. Heterogeneity estimates for the mixed-effect meta-analyses fitted per each climate variable (temperature vs precipitation), for each trait category (morphological vs phenological), using each path coefficient (column 'Relation') in the path diagram (Fig. 1a) as a response variable. *Q* is the total amount of heterogeneity and the column 'p-value' indicates whether this amount of heterogeneity is significant, I^2 reflects the proportion of the total heterogeneity due to between-study variance and ranges from 0 to 1.

Climate	Trait category	Relation	*Q*	p-value	I^2
Temperature	Morphological	CZ	1618.4	1.326E-270	0.956
Temperature	Morphological	ZG	249.0	1.025E-13	0.637
Temperature	Morphological	CG	251.9	1.756E-14	0.545
Temperature	Morphological	PG	345.8	1.8065E-27	0.730
Temperature	Morphological	CZG	124.0	0.070884	0.000
Temperature	Morphological	TotalCG	121.2	0.106066	0.286
Temperature	Phenological	CZ	1519.1	2.949E-258	0.960
Temperature	Phenological	ZG	146.8	0.000244	0.479
Temperature	Phenological	CG	121.9	0.014273	0.390
Temperature	Phenological	PG	287.6	5.357E-22	0.660
Temperature	Phenological	CZG	89.1	0.513577	0.062
Temperature	Phenological	TotalCG	55.6	0.998220	0.000
Precipitation	Morphological	CZ	1874.5	0	0.955
Precipitation	Morphological	ZG	257.9	3.717E-13	0.634
Precipitation	Morphological	CG	195.9	1.632E-06	0.429
Precipitation	Morphological	PG	411.1	3.497E-35	0.759
Precipitation	Morphological	CZG	112.3	0.466683	0.000
Precipitation	Morphological	TotalCG	153.6	0.005027	0.351
Precipitation	Phenological	CZ	1629.1	1.5E-279	0.951
Precipitation	Phenological	ZG	174.5	8.976E-07	0.528
Precipitation	Phenological	CG	136.1	0.001936	0.415
Precipitation	Phenological	PG	317.2	5.562E-26	0.680
Precipitation	Phenological	CZG	101.9	0.203374	0.059
Precipitation	Phenological	TotalCG	58.6	0.997642	0.000

Supplementary Table S11. Global across-study effect sizes obtained with mixed-effect meta-analytical models fitted per each climate variable (temperature vs precipitation), for each trait category (morphological vs phenological), using each path coefficient (column 'Relation') in the path diagram (Fig. 1a in the main text) as a response variable. Column 'Variable' specifies whether the global effect size for the relation is reported ('intrcpt') or if the estimated effect is reported for one of the covariates (Pvalue is the probability that the climate signal is spurious, i.e. $P_{\Delta AICc}$ from sliding window analyses, and WeathQ2 is weather quality variable, level "approximate", the reference level is "exact"; see Methods for more details). The performed tests were two-sided Wald tests.

Climate	Trait category	Rela-tion	Variable	Esti-mate	CI.lb	CI.ub	Chi2	p-value
Temperature	Morphology	CZ	intrcpt	0.079	-0.150	0.308	0.45	0.50160
Temperature	Morphology	CZ	WeathQ2	0.162	-0.062	0.386	4.05	0.13228
Temperature	Morphology	CZ	Pvalue	-0.299	-0.652	0.053	2.82	0.09312
Temperature	Morphology	ZG	intrcpt	0.049	-0.055	0.154	0.82	0.36504
Temperature	Morphology	CG	intrcpt	0.064	-0.067	0.194	1.08	0.29896
Temperature	Morphology	CG	WeathQ2	-0.084	-0.224	0.055	2.08	0.35411
Temperature	Morphology	CG	Pvalue	-0.052	-0.260	0.156	0.30	0.58325
Temperature	Morphology	PG	intrcpt	-0.679	-0.776	-0.583	217.4	3.34E-49
Temperature	Morphology	CZG	intrcpt	-0.017	-0.047	0.014	1.16	0.28236
Temperature	Morphology	CZG	WeathQ2	0.012	-0.016	0.040	1.44	0.48579
Temperature	Morphology	CZG	Pvalue	0.043	0.000	0.086	3.92	0.04758
Temperature	Morphology	TotalCG	intrcpt	0.064	-0.064	0.193	0.80	0.37014
Temperature	Morphology	TotalCG	WeathQ2	-0.050	-0.202	0.102	1.22	0.54328
Temperature	Morphology	TotalCG	Pvalue	-0.003	-0.193	0.187	0.01	0.92532
Temperature	Phenology	CZ	intrcpt	-0.366	-0.573	-0.158	12.46	0.00042
Temperature	Phenology	CZ	WeathQ2	0.054	-0.258	0.366	13.77	0.00102
Temperature	Phenology	CZ	Pvalue	0.533	0.196	0.871	10.08	0.00149
Temperature	Phenology	ZG	intrcpt	0.048	-0.026	0.121	1.64	0.20090
Temperature	Phenology	CG	intrcpt	-0.070	-0.164	0.023	2.34	0.12603
Temperature	Phenology	CG	WeathQ2	0.048	-0.085	0.181	2.45	0.29350
Temperature	Phenology	CG	Pvalue	0.002	-0.189	0.193	0.00	0.96856
Temperature	Phenology	PG	intrcpt	-0.526	-0.600	-0.452	199.6	2.55E-45
Temperature	Phenology	CZG	intrcpt	0.031	-0.007	0.069	3.23	0.07241
Temperature	Phenology	CZG	WeathQ2	-0.004	-0.057	0.050	3.24	0.19774
Temperature	Phenology	CZG	Pvalue	-0.040	-0.122	0.041	1.62	0.20315
Temperature	Phenology	TotalCG	intrcpt	-0.014	-0.078	0.051	0.17	0.67802
Temperature	Phenology	TotalCG	WeathQ2	0.032	-0.065	0.129	0.56	0.75439
Temperature	Phenology	TotalCG	Pvalue	0.011	-0.142	0.164	0.02	0.88437
Precipitation	Morphology	CZ	intrcpt	0.123	-0.114	0.361	1.02	0.31306
Precipitation	Morphology	CZ	WeathQ2	-0.031	-0.278	0.215	1.02	0.59987
Precipitation	Morphology	CZ	Pvalue	0.017	-0.347	0.380	0.01	0.91497
Precipitation	Morphology	ZG	intrcpt	0.086	-0.016	0.188	2.82	0.09327
Precipitation	Morphology	CG	intrcpt	0.050	-0.066	0.167	0.75	0.38699
Precipitation	Morphology	CG	WeathQ2	0.043	-0.066	0.152	1.75	0.41672
Precipitation	Morphology	CG	Pvalue	-0.158	-0.342	0.026	2.93	0.08697

Precipitation	Morphology	PG	intrcpt	-0.670	-0.789	-0.550	125.2	4.52E-29
Precipitation	Morphology	CZG	intrcpt	0.014	-0.013	0.040	1.00	0.31645
Precipitation	Morphology	CZG	WeathQ2	-0.009	-0.024	0.006	1.67	0.43312
Precipitation	Morphology	CZG	Pvalue	-0.009	-0.045	0.028	0.22	0.63701
Precipitation	Morphology	TotalCG	intrcpt	0.105	-0.031	0.241	2.29	0.13057
Precipitation	Morphology	TotalCG	WeathQ2	0.055	-0.093	0.204	3.35	0.18771
Precipitation	Morphology	TotalCG	Pvalue	-0.164	-0.357	0.029	2.63	0.10481
Precipitation	Phenology	CZ	intrcpt	-0.316	-0.571	-0.061	6.23	0.01257
Precipitation	Phenology	CZ	WeathQ2	-0.089	-0.380	0.202	9.91	0.00704
Precipitation	Phenology	CZ	Pvalue	0.517	0.108	0.926	6.43	0.01120
Precipitation	Phenology	ZG	intrcpt	0.065	-0.002	0.133	3.55	0.05963
Precipitation	Phenology	CG	intrcpt	0.075	-0.031	0.180	1.81	0.17855
Precipitation	Phenology	CG	WeathQ2	-0.023	-0.152	0.106	1.83	0.39998
Precipitation	Phenology	CG	Pvalue	-0.128	-0.308	0.053	1.81	0.17864
Precipitation	Phenology	PG	intrcpt	-0.531	-0.606	-0.456	197.2	8.51E-45
Precipitation	Phenology	CZG	intrcpt	-0.018	-0.059	0.023	0.47	0.49513
Precipitation	Phenology	CZG	WeathQ2	0.020	-0.032	0.071	0.63	0.73132
Precipitation	Phenology	CZG	Pvalue	0.015	-0.062	0.092	0.08	0.77300
Precipitation	Phenology	TotalCG	intrcpt	0.009	-0.067	0.086	0.06	0.80933
Precipitation	Phenology	TotalCG	WeathQ2	0.057	-0.038	0.151	1.58	0.45339
Precipitation	Phenology	TotalCG	Pvalue	-0.043	-0.198	0.112	0.30	0.58534

Supplementary Table S12. Results of one-sided binomial tests assessing whether the proportion of studies with positive CZG differed from randomly expected one, for each combination of climate and trait category. Column 'Mean prop of successes' reports the mean proportion of the studies with positive CZG, and the columns 'CI.lb' and 'CI.ub' report the 95% lower and upper confidence intervals, respectively.

Climate	Trait category	Number Successes	Number Trials	Mean prop of successes	CI.lb	CI.ub	p-value
Temperature	Morphology	51	106	0.481	0.398	1.00	0.68
Temperature	Phenology	55	93	0.602	0.501	1.00	0.048
Precipitation	Morphology	59	115	0.513	0.432	1.00	0.43
Precipitation	Phenology	41	95	0.411	0.326	1.00	0.97

SUPPLEMENTARY NOTES

Supplementary Note 1: List of sTraitChange Data Consortium members

Markus Ahola[1], Benjamin Letcher[2], Dirk Bauwens[3], Peter H. Becker[4], Katja Claus[3], Kirsten McDonnell[5], Steven H. Ferguson[6], Sandra Luque[7], Anne Goodenough[7], Paul Thompson[8], Richard King[9], Kristin Stanford[9], Peter Jones[9], Richard T. Holmes[10], Jen C. Rock[11], Philippe Pilard[12], Olof Olsson[13], Limoilou-Amelie Renaud[14], Lisa Schwanz[15]

[1]Department of Biology, University of Turku, FI-20014 Turku, Finland

[2]U.S. Geological Survey, Eastern Ecological Science Center, Silvio O. Conte Research Laboratory, One Migratory Way, Turners Falls, MA 01376

[3]Laboratory of Functional Morphology, Department of Biology, University of Antwerp, Universiteitsplein 1, 2610 Wilrijk, Belgium

[4]Institute of Avian Research, An der Vogelwarte 21, D-26386 Wilhelmshaven, Germany

[5]U.S. Fish and Wildlife Service, Division of Migratory Birds, Albuquerque, New Mexico, USA

[6]Fisheries and Oceans Canada, Winnipeg, MB, Canada

[7]School of Natural and Social Sciences, University of Gloucestershire, Cheltenham, UK[8]University of Aberdeen, School of Biological Sciences, Lighthouse Field Station, George Street, Cromarty, IV11 8YL, UK.

[9]Department of Biological Sciences, Northern Illinois University, DeKalb, Illinois, USA

[10]Department of Biological Sciences, Dartmouth College, Hanover, NH 03755 USA

[11]Environment and Climate Change Canada, 17 Waterfowl Lane, Sackville, New Brunswick, E4L 4N1, Canada

[12]Ligue de Protection des Oiseaux (LPO), France, 8 rue Maurice Bonnafoux, 13200 Arles, France

[13]Stockholm Resilience Centre, Stockholm University, Sweden

[14]Université de Sherbrooke, 2500 boulevard de l'Université, Sherbrooke, Quebec J1K 2R1, Canada

[15]Evolution & Ecology Research Centre, School of Biological, Earth, and Environmental Sciences, UNSW Sydney, Sydney, NSW 2052, Australia

Supplementary Note 2: Other combinations of climate variables and traits

We caution that for the studies on combinations of morphology and precipitation, morphology-temperature and phenology-precipitation, the probability that the climate signal was spurious is non-negligible for a large proportion of the studies, therefore these results should be interpreted carefully. Similar to the observed high heterogeneity in responses of phenology to temperature$_d$, we also found heterogeneous responses of phenology to precipitation$_d$ and of morphology to either year-detrended climate variable (Supplementary

Table S10). The average across-study effects were not statistically significant for any of these trait-climate combinations (Supplementary Table S11, Supplementary Figs. S27-S29).

For all of these combinations of trait category and climate variable the proportion of the studies with positive CZG did not significantly differ from the expected by chance only (Supplementary Table S12). The degree of phylogenetic structuring differed for the models fitted to different paths (CZ, ZG, CZG and CG, see Suppl. Figs S22-S25) as well as between the models fitted to the full dataset or to the subsets of birds only and mammals only.

Supplementary Note 3: Variance partitioning in SEM

Our variance partitioning applied to the "population growth model" in our SEM (Supplementary Note 8: Variance partitioning) showed that population size explained the largest proportion of variation in G (median = 0.26, 5th percentile = 0.01, 95th percentile =0.65 across studies, Supplementary Fig. S22). Phenology explained a lower proportion of the variation in G, although in some models it was quite high (median = 0.05, 5th percentile = 0.001, 95th percentile = 0.43 across studies). Finally, the direct effect of $temperature_d$ explained a median 0.03 of the variation in G across the models (5th percentile = 0.001, 95th percentile = 0.42). The contribution of temperature to population growth rate is only partial here, as it only captures the direct effect of $temperature_d$ on G (i.e. we applied variance partitioning to G). The effect of phenology on G includes an indirect effect of $temperature_d$ (CZG pathway) and we note that a large proportion of variation in Z was explained by C (see R^2 of the "trait model" within our SEM, Supplementary Fig. S20).

Supplementary Note 4: Phylogenetic signal

When re-analysing the data for birds and reptiles separately, Pagel's λ was not distinguishable from 0 in most cases (Supplementary Figs. S23-S26). Thus, phylogenetic signal was not distinguishable from no signal for the model focusing on phenological responses to temperature$_d$, CZ (birds, mean lambda across 100 randomly drawn posterior phylogenies = 0.04; min = 0, max = 0.11; mammals lambda = 0.02; 0, 0.92), for the model focusing on effects of phenology on population growth rate (birds λ = 0.09; min = 0, max = 0.32; mammals λ = 1; 1, 1), for the model focusing on the phenology-mediated effect of temperature on population growth rate (birds λ = 0; min = 0, max = 0; mammals λ could not be estimated for the dataset with 7 points only), and for the model focusing on the direct effect of temperature$_d$ on population growth rate, not mediated by phenology (birds λ = 0; min = 0, max = 0; mammals λ could not be estimated for the dataset with 7 points only).

Supplementary Note 5: Climatic window durations

We decided to not apply any minimum threshold on the climatic window duration because no *a priori* knowledge on what the realistic minimum window duration exists for such diverse species and locations. Indeed, the studies that systematically identified appropriate climatic windows so far were conducted for passerines mainly, focusing on phenology (McLean et al., 2016) and on body condition (McLean et al., 2018; McLean et al., 2020). And, although (McLean et al., 2018) used a minimum window duration of 5 days, they have found that windows of 10 days were selected as appropriate for some species. In our study, which relies on weekly temporal resolution, the duration of such windows would likely be identified as 1 week, and if we had decided to exclude windows of 1 week duration, we would exclude potentially true climate windows. Furthermore, importance of the extreme weather events for many species may be represented by such rather short window durations.

And implications of such short-term extreme weather periods for demography may be important (Shipley et al., 2020).

We thus tested for all possible windows with the minimum window duration being 1 week and the maximum being 108 weeks, over a period of two years before the so-called reference day (the value of which was study-specific, see Methods in the main text). We explored all possible beginnings for the climate windows at all possible lags from this reference day.

After the climatic windows were identified with *climwin*, we removed studies in the dataset with window durations of more than 51 weeks (1 and 8 studies for precipitation and temperature, respectively) from the subsequent analyses, as such long windows were a priori considered to be unrealistic. The median window duration across the studies was 3 and 2 weeks for precipitation and temperature, respectively (Supplementary Fig. S17).

Supplementary Note 6: Testing for non-linearity in relations

We visually expected relations between year-detrended climate variable (temperature and precipitation) and trait (phenology and morphology) for each study. The majority of the relations were linear according to the visual inspection of scatterplots. To statistically confirm that linear relations are meaningful we fitted mixed-effects models per each combination of trait category (phenology and morphology) and climate variable (temperature and precipitation). To test the linearity assumption between climate and traits, we used trait as a response variable and included both linear and quadratic effects of climate as fixed quantitative predictors in the model. To test the linearity assumption between population growth and traits, we used population growth rate as a response variable and included both linear and quadratic effects of traits as fixed quantitative predictors. In both models, we included study as a random intercept and modelled temporal autocorrelation as an

autoregressive process of the first order. We assessed the significance of the quadratic effect (of climate and of traits, for each type of the model, respectively) with Likelihood Ratio Tests that compared the model with a quadratic effect to that without the quadratic effect. Since these models assumed that the fitted quadratic shape is the same across all studies (which is rather unlikely), we also fitted the same models by including study as a random slope in the model (in fact these are very similar to the models that are tested in our SEMs, where, for example, the relation between temperature and phenology is allowed to vary in each study and we then use meta-analysis to estimate the across-study slope CZ). All models were fitted with spaMM R package version 4.4.16 (Rousset & Ferdy, 2014). As for the mixed-effects models described in the main text, we z-transformed both the traits and population growth rate prior to fitting the models. Our results support linear relations in most cases (Supplementary Table S9).

Supplementary Note 7: Sources of species-specific characteristics

We obtained the data on species generation time of birds from (Bird et al., 2020) and for other species mainly from the IUCN Red List website (https://www.iucnredlist.org/). Diet categories are taken from EltonTraits 1.0 database (Wilman et al., 2014) and were grouped so that the groups 'Herbivore' and 'PlantSeed' were included as 'herbivore' in our analyses; 'Invertebrate', 'Insectivore', 'Carnivore', 'VertFish', 'VertFishScav', 'VertInvertEggs', and 'InvertFish' were all grouped under 'carnivore' in our analyses, and 'omnivore' was considered as 'omnivore'. The data for migratory mode was mainly taken from the Sibly dataset (Sibly et al., 2012). We tried to fill in the gaps as far as possible by searching species-specific literature.

Supplementary Note 8: Variance partitioning

We partitioned the variance in population growth rate (G) that was explained by temperature, phenology and population size in the "population growth model" (see Methods, "Trait-mediated effects of climate on G"), one of the models constituting our SEMs for each study. To partition the variance, we used R^2_{lik} that was proposed by (Ives, 2019) and is available in the R package *rr2* as function R2_lik(). R^2_{lik} is based on the likelihood of fitted models and thus reflects the amount of information that the model contains. It also has favourable properties compared to two other metrics proposed by (Ives, 2019), e.g. it cannot be negative for any of the predictors included in the model.

REFERENCES

Bird, J. P., Martin, R., Akçakaya, H. R., Gilroy, J., Burfield, I. J., Garnett, S. T., Symes, A., Taylor, J., Şekercioğlu, Ç. H., & Butchart, S. H. M. (2020). Generation lengths of the world's birds and their implications for extinction risk. *Conservation Biology, 34*(5). https://doi.org/10.1111/cobi.13486

Ives, A. R. (2019). R 2 s for Correlated Data: Phylogenetic Models, LMMs, and GLMMs. *Systematic Biology*, *68*(2). https://doi.org/10.1093/sysbio/syy060

McLean, N., Lawson, C. R., Leech, D. I., & van de Pol, M. (2016). Predicting when climate-driven phenotypic change affects population dynamics. *Ecology Letters*, *19*(6), 595–608. https://doi.org/10.1111/ele.12599

McLean, N. M., van der Jeugd, H. P., van Turnhout, C. A. M., Lefcheck, J. S., & van de Pol, M. (2020). Reduced avian body condition due to global warming has little reproductive or population consequences. *Oikos*, *129*, 714–730. https://doi.org/10.1111/oik.06802

McLean, N., Van Der Jeugd, H. P., & Van De Pol, M. (2018). High intra-specific variation in avian body condition responses to climate limits generalisation across species. *PLoS ONE*, *13*(2), e0192401. https://doi.org/10.1371/journal.pone.0192401

Radchuk, V., Reed, T., Teplitsky, C., van de Pol, M., Charmantier, A., Hassall, C., Adamík, P., Adriaensen, F., Ahola, M. P., Arcese, P., Miguel Avilés, J., Balbontin, J., Berg, K. S., Borras, A., Burthe, S., Clobert, J., Dehnhard, N., de Lope, F., Dhondt, A. A., … Kramer-Schadt, S. (2019). Adaptive responses of animals to climate change are most likely insufficient. *Nature Communications*, *10*, 3109. https://doi.org/10.1038/s41467-019-10924-4

Rousset, F., & Ferdy, J.-B. (2014). Testing environmental and genetic effects in the presence of spatial autocorrelation. *Ecography Journal*, *37*(December 2013), 781–790. https://doi.org/10.1111/ecog.00566

Shipley, J. R., Twining, C. W., Taff, C. C., Vitousek, M. N., Flack, A., & Winkler, D. W. (2020). Birds advancing lay dates with warming springs face greater risk of chick mortality. *Proceedings of the National Academy of Sciences of the United States of America*, *117*(41), 25590–25594. https://doi.org/10.1073/pnas.2009864117

Sibly, R. M., Witt, C. C., Wright, N. A., Venditti, C., Jetz, W., & Brown, J. H. (2012). Energetics, lifestyle, and reproduction in birds. *Proceedings of the National Academy of Sciences of the United States of America*, *109*(27), 10937–10941. https://doi.org/10.1073/pnas.1206512109

Wilman, H., Belmaker, J., Simpson, J., de la Rosa, C., Rivadeneira, M. M., & Jetz, W. (2014). EltonTraits 1.0: Species-level foraging attributes of the world's birds and mammals. *Ecology*, *95*(7), 2027. https://doi.org/10.1890/13-1917.1
