# Supplementary material for: Changes in phenology mediate vertebrate population responses to temperature globally
Source: Nat Commun. 2026 Jan 12;17:479. doi: 10.1038/s41467-025-68172-8 (PMC12800269; doi:10.1038/s41467-025-68172-8)
Supplement: Supplementary file 2 — Description of Additional Supplementary File [file 41467_2025_68172_MOESM2_ESM.pdf]

Description of Additional Supplementary File

File Name: Supplementary Data 1

Description: Overview of the studies assembled for the meta-analysis.
