# Supplementary material for: Changes in phenology mediate vertebrate population responses to temperature globally
Source: Nat Commun. 2026 Jan 12;17:479. doi: 10.1038/s41467-025-68172-8 (PMC12800269; doi:10.1038/s41467-025-68172-8)
Supplement: Supplementary file 4 — Reporting Summary [file 41467_2025_68172_MOESM4_ESM.pdf]

nature portfolio

Corresponding author(s): Viktoriia Radchuk
Last updated by author(s): 17.11.2025

Reporting Summary

Nature Portfolio wishes to improve the reproducibility of the work that we publish. This form provides structure for consistency and transparency in reporting. For further information on Nature Portfolio policies, see our Editorial Policies and the Editorial Policy Checklist.

Statistics

For all statistical analyses, confirm that the following items are present in the figure legend, table legend, main text, or Methods section.

n/a	Confirmed	
☐	☒	The exact sample size (*n*) for each experimental group/condition, given as a discrete number and unit of measurement
☐	☒	A statement on whether measurements were taken from distinct samples or whether the same sample was measured repeatedly
☐	☒	The statistical test(s) used AND whether they are one- or two-sided<br>*Only common tests should be described solely by name; describe more complex techniques in the Methods section.*
☐	☒	A description of all covariates tested
☐	☒	A description of any assumptions or corrections, such as tests of normality and adjustment for multiple comparisons
☐	☒	A full description of the statistical parameters including central tendency (e.g. means) or other basic estimates (e.g. regression coefficient) AND variation (e.g. standard deviation) or associated estimates of uncertainty (e.g. confidence intervals)
☐	☒	For null hypothesis testing, the test statistic (e.g. *F*, *t*, *r*) with confidence intervals, effect sizes, degrees of freedom and *P* value noted<br>*Give P values as exact values whenever suitable.*
☒	☐	For Bayesian analysis, information on the choice of priors and Markov chain Monte Carlo settings
☐	☒	For hierarchical and complex designs, identification of the appropriate level for tests and full reporting of outcomes
☐	☒	Estimates of effect sizes (e.g. Cohen's *d*, Pearson's *r*), indicating how they were calculated

Our web collection on statistics for biologists contains articles on many of the points above.

Software and code

Policy information about availability of computer code

Data collection	We performed systematic literature review to identify the studies that satisfy our criteria. We next extracted the data from the papers that were retained by either manually extracting the data whenever possible, or by digitising the plots with WebPLotDigitizer v4.8 (Ankit Rohatgi): https://apps.automeris.io/wpd4/ . For those studies from which we could not extract the raw data from the papers, we contacted the authors by email asking to share their data. The sTraitChange dataset assembled in this study has been deposited on Zenodo as part of the R project 'sTraitChange_Analyses' under https://doi.org/10.5281/zenodo.17629266
Data analysis	All statistical analyses were performed in R statistical software v4.4.1. To ensure reproducibility of this study, we have developed an R package sTraitChange v1.1.0, deposited on Zenodo: https://doi.org/10.5281/zenodo.17629295 .This package contains all the custom-made functions needed for running climate sliding window analyses, structural equation modelling and meta-analyses. We also publicly share the whole work-flow as an R project that includes all R scripts that were used in this study: sTraitChange Analyses & Data v1.0.0, deposited on Zenodo: https://doi.org/10.5281/zenodo.17629266.

For manuscripts utilizing custom algorithms or software that are central to the research but not yet described in published literature, software must be made available to editors and reviewers. We strongly encourage code deposition in a community repository (e.g. GitHub). See the Nature Portfolio guidelines for submitting code & software for further information.

Data

Policy information about availability of data

All manuscripts must include a data availability statement. This statement should provide the following information, where applicable:

- Accession codes, unique identifiers, or web links for publicly available datasets
- A description of any restrictions on data availability
- For clinical datasets or third party data, please ensure that the statement adheres to our policy

The sTraitChange dataset generated in this study has been deposited on Zenodo as part of the R project 'sTraitChange_Analyses' under https://doi.org/10.5281/zenodo.17629266 90. See the Readme of the R project 'sTraitChange_Analyses' for more information. The overview of the dataset is provided as the Supplementary Data 1.

Research involving human participants, their data, or biological material

Policy information about studies with human participants or human data. See also policy information about sex, gender (identity/presentation), and sexual orientation and race, ethnicity and racism.

Reporting on sex and gender	Gender-based analyses were not performed because this study is not focusing on humans but is a meta-analysis of the published research that collected observational data on wild-living vertebrates. Whenever the original studies investigated the effect of sex on phenotypic trait or on the relation between the trait and the climate and phenotypic traits were reported per sex, we had included such data disaggregated per sex in our dataset (by including separate study IDs for different sexes).
Reporting on race, ethnicity, or other socially relevant groupings	No socially-constructed or socially-relevant categorization variables were used because our study is a meta-analysis of the published data on wild invertebrates (excluding humans)
Population characteristics	Not applicable as we did not have human research participants
Recruitment	Not applicable as we did not have human research participants
Ethics oversight	Not applicable as we did not have human research participants

Note that full information on the approval of the study protocol must also be provided in the manuscript.

Field-specific reporting

Please select the one below that is the best fit for your research. If you are not sure, read the appropriate sections before making your selection.

☐ Life sciences ☐ Behavioural & social sciences ☒ Ecological, evolutionary & environmental sciences

For a reference copy of the document with all sections, see nature.com/documents/nr-reporting-summary-flat.pdf

Ecological, evolutionary & environmental sciences study design

All studies must disclose on these points even when the disclosure is negative.

Study description	In this study we addressed the question "To what extent the effects of climate on phenotypic traits propagate to affect population growth rate?". We performed a systematic literature review to extract relevant studies from the published literature. Our assembled dataset consists of 213 studies covering four vertebrate classes (extracted from 73 papers). For each of these studies we performed climatic window analysis to extract two climate variables: temperature and precipitation. Climatic window analyses allow finding the temporal window over which a specific climate variable explains the variation in the studied phenotypic traits the best. We then analysed each of the studies by using Structural Equation Modelling (SEM), to assess the effects of climate on traits, and of traits on population growth rates, as well as the effect of the climate variable on the population growth rate that is mediated by the phenotypic trait. In our SEM we also included population size, to account for its potential confounding effects. Once these effects (also called "path coefficients") were extracted from each study, we performed a meta-analysis on them. Our meta-analysis accounted for phylogenetic relatedness between species and included as random intercepts following terms: the study, the location, and the species. We also weighted the residual variation in our meta-analytical mixed-effects model by the inverse of the squared standard errors of the estimated path coefficients to propagate the uncertainty from SEMs.
Research sample	This study is based on the systematic literature review and thus the dataset used in this study consists of the (mainly) published data. Our assembled dataset consists of 213 studies (a study is defined as a unique combination of the species, the location and the phenotypic trait studied) that cover four vertebrate classes worldwide. Morphological traits were represented by 116 and phenological traits by 97 studies. The dataset was dominated by studies on birds, followed by reptiles, mammals and fish. The subset on morphological traits was less skewed towards the birds compared to the subset focusing on phenological traits (exact numbers of studies per phenotypic trait and taxon are specified on the inset to Figure 2). The majority of the extracted studies were conducted in the northern hemisphere.

Sampling strategy	We performed a systematic literature review aimed at uncovering as many studies as possible that would allow us addressing our research questions across species and habitats. We thus did not perform sample-size calculation to guide our study design, as would be typical in classical experimental studies.
Data collection	The screening of the abstracts returned by the systematic literature review was conducted by eight of the co-authors (VR, CVJ, GC, EM, TER, JC, SK, and NMcL). Two coauthors (CVJ and VR) then extracted all the relevant data from the 73 papers that satisfied all selection criteria. Whenever possible we extracted the raw data from the published papers directly (from the table or a figure- by using WebPlotDigitizer). If this was not the case, we have contacted the authors of the original published study and asked them to share the data.
Timing and spatial scale	Since this study is based on the results of a systematic literature review, the period of data collection covers those periods reported in the original papers. Our dataset spans studies with duration of minimum 9 years and up to 62 years. The median study duration across the studies focusing on phenological traits was 25 years and across the studies focussing on morphological traits - 14.5 years. The earliest study in our dataset started in 1953 with the median starting year across all studies being 1994. The study with the latest start in our dataset started the data recording in 2011. The median of the last study year across the studies was 2014, with the last year for the study that ended the earliest being 1986 and the last year for the study that ended most recently being 2019.<br>Spatial scale: over the globe
Data exclusions	We had specified a set of criteria based on which the study was included in our dataset, as follows:<br>- the study duration should be at least 9 years<br>- the study should measure a quantitative phenotypic trait and population size yearly and focus on impacts of some climate variable on the trait and / or demography. As phenotypic traits we considered two groups: phenological and morphological traits. Phenological traits are those that reflect the timing of recurring biological events such as reproduction and migration, for example the day of return of migrants, the day when the eggs are laid or the rut day. Morphological traits reflect measurements of the body mass, size or shape, for example wing length, tarsus length, body mass.<br>- the study focused on a vertebrate species
Reproducibility	As we have not performed original data collection, we cannot speak about reproducibility of the observational studies on which our meta-analysis relies. However, we ensured the reproducibility of our findings by sharing the data and the complete workflow needed for the analyses on Zenodo: sTraitChange Analyses & Data v1.0.0. https://doi.org/10.5281/zenodo.17629266 (2025). We have also made available the custom-made functions needed to perform the analyses as R package sTaitChange: sTraitChange R package v 1.1.0. https://doi.org/10.5281/zenodo.17629295 (2025).
Randomization	Not relevant as this study is based on the (mainly) published studies extracted based on the systematic literature review.
Blinding	Not relevant because this study is based on previously published studies that were collected via systematic literature review.

Did the study involve field work? ☐ Yes ☒ No

Reporting for specific materials, systems and methods

We require information from authors about some types of materials, experimental systems and methods used in many studies. Here, indicate whether each material, system or method listed is relevant to your study. If you are not sure if a list item applies to your research, read the appropriate section before selecting a response.

Materials & experimental systems

n/a	Involved in the study
☒	☐ Antibodies
☒	☐ Eukaryotic cell lines
☒	☐ Palaeontology and archaeology
☐	☒ Animals and other organisms
☒	☐ Clinical data
☒	☐ Dual use research of concern
☒	☐ Plants

Methods

n/a	Involved in the study
☒	☐ ChIP-seq
☒	☐ Flow cytometry
☒	☐ MRI-based neuroimaging

Animals and other research organisms

Policy information about studies involving animals; ARRIVE guidelines recommended for reporting animal research, and Sex and Gender in Research

Laboratory animals	The study did not involve laboratory animals
Wild animals	We did not directly work with wild animals but the original published studies that were used in our meta-analyses collected data on wild animals. Our dataset contains data on 74 invertebrate species, mainly birds but also a fair number of reptiles (especially for studies on morphological traits) and mammals.

Reporting on sex	Whenever the original studies investigated the effect of sex on phenotypic trait or on the relation between the trait and the climate and phenotypic traits were reported per sex, we had included such data disaggregated per sex in our dataset (by including separate study IDs for different sexes).
Field-collected samples	The study did not involve samples collected from the field
Ethics oversight	No ethical approval was needed because our study is based on a systematic literature review, we thus have not gather primary data ourselves.

Note that full information on the approval of the study protocol must also be provided in the manuscript.

Plants

Seed stocks	Not applicable, our study is a meta-analysis of the published data on wild invertebrates
Novel plant genotypes	Not applicable, our study is a meta-analysis of the published data on wild invertebrates
Authentication	Not applicable, our study is a meta-analysis of the published data on wild invertebrates
